# *In Vivo* Therapy with M2e-Specific IgG Selects for an Influenza A Virus Mutant with Delayed Matrix Protein 2 Expression

**DOI:** 10.1128/mBio.00745-21

**Published:** 2021-07-13

**Authors:** Silvie Van den Hoecke, Marlies Ballegeer, Bram Vrancken, Lei Deng, Emma R. Job, Kenny Roose, Bert Schepens, Lien Van Hoecke, Philippe Lemey, Xavier Saelens

**Affiliations:** a VIB-UGent Center for Medical Biotechnology, VIB, Ghent, Belgium; b Department of Biomedical Molecular Biology, Ghent University, Ghent, Belgium; c Department of Biochemistry and Microbiology, Ghent University, Ghent, Belgium; d KU Leuven—University of Leuven, Department of Microbiology, Immunology and Transplantation, Rega Institute for Medical Research, Clinical and Epidemiological Virology, Leuven, Belgium; e VIB-UGent Center for Inflammation Research, VIB, Ghent, Belgium; Icahn School of Medicine at Mount Sinai

**Keywords:** influenza, broadly protective, deep sequencing, escape, immunotherapy, monoclonal antibodies

## Abstract

The ectodomain of matrix protein 2 (M2e) of influenza A viruses is a universal influenza A vaccine candidate. Here, we report potential evasion strategies of influenza A viruses under *in vivo* passive anti-M2e IgG immune selection pressure in severe combined immune-deficient (SCID) mice. A/Puerto Rico/8/34-infected SCID mice were treated with the M2e-specific mouse IgG monoclonal antibodies (MAbs) MAb 65 (IgG2a) or MAb 37 (IgG1), which recognize amino acids 5 to 15 in M2e, or with MAb 148 (IgG1), which binds to the invariant N terminus of M2e. Treatment of challenged SCID mice with any of these MAbs significantly prolonged survival compared to isotype control IgG treatment. Furthermore, M2e-specific IgG2a protected significantly better than IgG1, and even resulted in virus clearance in some of the SCID mice. Deep sequencing analysis of viral RNA isolated at different time points after treatment revealed that the sequence variation in M2e was limited to P10H/L and/or I11T in anti-M2e MAb-treated mice. Remarkably, in half of the samples isolated from moribund MAb 37-treated mice and in all MAb 148-treated mice, virus was isolated with a wild-type M2 sequence but with nonsynonymous mutations in the polymerases and/or the hemagglutinin genes. Some of these mutations were associated with delayed M2 and other viral gene expression and with increased resistance to anti-M2e MAb treatment of SCID mice. Treatment with M2e-specific MAbs thus selects for viruses with limited variation in M2e. Importantly, influenza A viruses may also undergo an alternative escape route by acquiring mutations that result in delayed wild-type M2 expression.

## INTRODUCTION

Human influenza is a contagious respiratory disease and a major cause of morbidity and mortality, despite the implementation of annual influenza vaccination campaigns and the use of antivirals. Licensed influenza vaccines protect primarily by inducing neutralizing antibodies against hemagglutinin (HA), the major membrane protein of influenza A and B viruses (1). However, the efficacy of these vaccines is limited due to their strain specificity and the possibility of influenza A and B viruses undergoing antigenic drift (2). By targeting more conserved antigenic regions of the virus, influenza vaccines could offer protection against disease caused by antigenically drifted viruses or even against multiple influenza A virus subtypes (3). For example, the protective potential of vaccines that are based on the conserved ectodomain of matrix protein 2 (M2e) has been explored extensively (4–7).

M2 is a 97-amino-acid (aa)-residue homotetrameric membrane protein of influenza A viruses with selective ion channel activity that exerts multiple functions in the infected host cell, including contributions to virus uncoating, inflammasome activation, and subversion of the host autophagy machinery (8–12). In contrast to HA and neuraminidase (NA), a relatively small number of M2 molecules (20 to 60 per virion) are present in the influenza virion (13–15). On the other hand, M2 is highly expressed on the plasma membrane of infected cells (16, 17). M2e is 23 amino acid residues long, and its sequence is conserved between human and avian, as well as swine, influenza viruses (18). This sequence conservation of M2e can be explained by the very limited immune pressure imposed by the host immune system against M2e and the overlap of its coding information with the open reading frame of M1, one of the most conserved influenza proteins (19, 20). In fact, the first 9 amino acid residues of M2 are almost absolutely conserved in all influenza A virus strains, even in H17N10 and H18N11 viruses that were recently isolated from bats (21).

Influenza viruses can escape from natural and vaccine-induced immune pressure as a consequence of the relatively high error rate (2.3 × 10^−5^ misincorporations per nucleotide position per genome replication) of their RNA-dependent RNA polymerase, together with the high mutational tolerability of the dominant antigenic loops in their HA and NA proteins (22–24). The mechanisms of influenza virus escape from monoclonal and polyclonal antibodies that target HA can be recapitulated *in vitro* and have been extensively studied (1–3, 25, 26). Viruses that can escape from *in vitro* inhibition by a broadly neutralizing HA stalk-specific monoclonal antibody have been described previously (27). These viruses escaped by acquiring a mutation that either prevented antibody binding or resulted in enhanced HA-mediated membrane fusion activity (27).

In general, M2e-specific antibodies do not restrict the *in vitro* growth of influenza A viruses, although a few laboratory strains are susceptible (28). Instead, M2e-specific IgG antibodies protect by recruiting immune cells such as alveolar macrophages and natural killer cells to the infected cells through activating Fcγ receptor (FcγR) engagement (29–31). Nevertheless, anti-M2e IgG escape mutants have been isolated by *in vitro* selection of A/Udorn/72 virus, a strain that is sensitive *in vitro* to the direct action of anti-M2e IgG (16). In most of these escape viruses, resistance to anti-M2e IgG was linked to a single-amino-acid-residue change in the cytoplasmic domain of M2 or the N terminus of M1 (16). In one instance, resistance was acquired by a glutamic acid to glycine residue mutation at position 8 in M2e (and M1), which resulted in loss of anti-M2e IgG binding (16). In a second study, severe combined immune-deficient (SCID) mice were treated with anti-M2e monoclonal antibodies (MAbs) prior to infection with PR8 virus (32). In 65% of these mice, escape viruses emerged that carried a proline to histidine or leucine residue substitution at position 10 in M2 (32). Although only the M2e sequence was thoroughly investigated in this study, 6 synonymous and 2 nonsynonymous mutations (A127T and V228L) were also detected in M1 (32). However, these mutations were based on viral sequence analysis of virus from a lung sample after amplification on MDCK cells. Therefore, part of the *in vivo* viral diversity may have been missed due to the amplification step in cell culture. In addition, sequence analysis of the MDCK-grown virus was performed by direct Sanger sequencing of purified reverse transcription-PCR (RT-PCR) product (32). Although state of the art in 2005, this method has a relatively limited sensitivity. Furthermore, all anti-M2e MAbs used in that study were of the IgG2a isotype and recognized a similar epitope. It is now clear that the B-cell response elicited by M2e-based vaccines is oligoclonal, and M2e-specific MAbs that bind to different parts of M2e have been described previously (33–35). Moreover, FcγRs play a very important role in anti-M2e-based immunity and these receptors differentially engage different antibody isotypes (29, 30, 36, 37). Therefore, it is important to explore the possible escape routes of influenza A viruses from selection imposed by different anti-M2e IgG isotypes and to do so *in vivo*.

Here, we used a whole-influenza-virus-genome deep sequencing approach to explore the possible escape routes of influenza viruses in SCID mice under anti-M2e MAb pressure (38). This approach allows genome-wide analysis with higher sensitivity than that of Sanger sequencing of PCR products, and thus may allow the identification of minor variants in the viral population. To investigate how influenza A virus evolves within a mammalian host under the immune pressure of anti-M2e IgG, we treated PR8-infected SCID mice with one of three well-defined anti-M2e IgG MAbs and performed influenza virus genome-wide deep sequencing analysis on bronchoalveolar lavage (BAL) fluid isolated at different time points after infection. Anti-M2e Mab treatment significantly prolonged survival and, as anticipated, viruses with an altered M2e epitope emerged in the course of the infection. Interestingly, we also found that virus populations with a combination of mutations in PB2, PA, and HA but with wild-type M2 sequences emerged in some of the treated SCID mice. These mutations were associated with delayed expression of viral proteins, including that of M2 in infected cells, reduced *in vitro* growth kinetics, and increased resistance to anti-M2e MAb therapy *in vivo*.

## RESULTS

### Treatment with anti-M2e MAbs significantly prolongs survival of challenged SCID mice.

**FIG 1:**
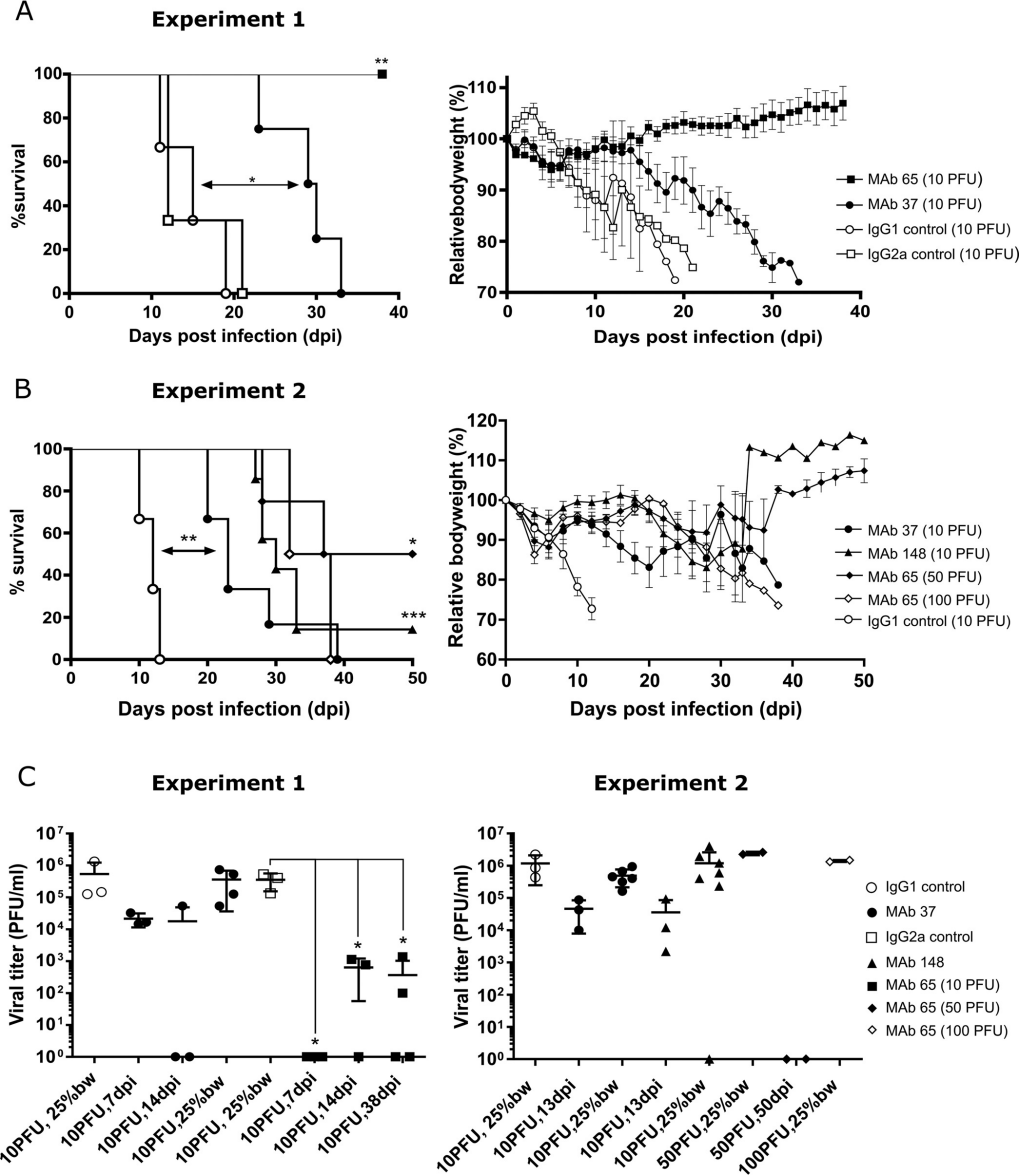
Anti-M2e monoclonal antibodies (MAbs) significantly prolong survival in severe combined immune-deficient (SCID) mice and reduce lung viral titer. One day before infection, SCID mice were intraperitoneally (i.p.) injected with 100 μg of MAb 37, MAb 65, MAb 148, or negative-control MAbs, and at weekly intervals thereafter with 50 μg. At 24 h after the first MAb injection, mice were challenged with 10 PFU of PR8 virus (in experiment 2, two groups of mice treated with MAb 65 were infected with either 50 [*n* = 4] or 100 PFU [*n* = 2], as shown in panel B). Survival (left) and body weight (right) were monitored daily. In the body weight graphs, data points represent averages and error bars standard deviations. (A) Treatment with MAb 65 significantly prolongs survival compared to that of all other groups (*n* = 4/group; **, *P* = 0.0067). Treatment with MAb 37 significantly prolongs survival compared to that of control-treated groups (MAb 37, *n* = 4; *, *P* = 0.0101; IgG1 and IgG2a controls, *n* = 3). (B) Treatment with MAb 37, MAb 148, and MAb 65 (when infected with 50 PFU) significantly prolongs survival compared to that of control-treated groups. MAb 37 (*n* = 6); **, *P* = 0.0018. MAb 148 (*n* = 7); ***, *P* = 0.0008. MAb 65 (*n* = 4); *, *P* = 0.0101. IgG1 control (*n* = 3). The survival between groups was compared using the log rank test. (C) The viral titer in bronchoalveolar lavage (BAL) fluid was determined by viral plaque titration on MDCK cells. Left and right graphs represent data obtained from independently performed experiments. (Left) Viral titer in MAb 65-treated mice was significantly different (*, *P* < 0.05; unpaired *t* test) from the IgG2a control group after infection with 10 PFU at 7 days postinfection (dpi) (*P* = 0.0378), 14 dpi (*P* = 0.0380), and at the end of the experiment (38 dpi; *P* = 0.0148). (Right) Differences in viral titer between mice treated with M2e-specific or control MAbs were not statistically significant (*P* > 0.05; unpaired *t* test). One mouse treated with MAb 148 was still alive at 50 dpi, was sacrificed on this day, and had no detectable viral titer. BAL fluid from MAb 65-treated mice infected with 50 PFU was isolated at 50 dpi (*n* = 2, no virus detected) or when mice had lost at least 25% of their initial body weight (bw) (*n* = 2, 25% bw).

SCID mice were infected with a low dose of a well-characterized plasmid-derived PR8 virus, which we had previously characterized genetically by deep sequencing (38). We reasoned that in the absence of an adaptive immune response against the challenge virus, anti-M2e IgG treatment would prolong survival and result in a chronic influenza A virus infection, which could favor the emergence of anti-M2e IgG escape mutants. Prior to and weekly after infection, mice received an intraperitoneal (i.p.) injection of one of three different well-characterized anti-M2e or isotype control MAbs. MAb 37 (IgG1) and MAb 65 (IgG2a) bind with similar affinity to a part of M2e that involves residues 5 to 15, while MAb 148 (IgG1) binds the highly conserved N-terminal 8-aa residues of M2e (30, 33, 34). In a first experiment, SCID mice were challenged with 10 PFU of the virus, a dose that was lethal for the control MAb-treated mice (Fig. 1A). All mice that had been challenged with 10 PFU of virus and treated with MAb 65 survived and displayed limited, transient body weight loss early after infection. MAb 37 treatment significantly increased the survival time of the challenged SCID mice compared with that under isotype control antibody treatment (Fig. 1A). In a second experiment, we increased the challenge dose to 50 and 100 PFU of PR8 virus for the MAb 65 group, and again used 10 PFU to challenge the MAb 37-, MAb 148-, and isotype control-treated mice. Two out of 4 mice in the MAb 65 group that had been challenged with 50 PFU of the PR8 virus were still alive at 50 days postinfection, whereas the other 2 mice in this group had to be euthanized on days 28 and 37 after challenge (Fig. 1B, experiment 2). All MAb 65-treated mice that had been challenged with 100 PFU of PR8 virus had reached the ethical endpoint by day 38 after infection (Fig. 1B). Treatment with MAb 37 or MAb 148 resulted in a comparable prolongation of survival, which was significantly different compared to that in the isotype control treatment (Fig. 1B). We conclude that treatment with anti-M2e MAbs significantly prolongs survival of PR8-challenged SCID mice compared to that of isotype control-treated animals (Fig. 1A and B).

### MAb 65 treatment of PR8-infected SCID mice restricts viral replication.

M2e-based vaccination strategies result in reduced lung viral loads in immunocompetent mice (4, 30). To assess if this was also the case in the passively treated PR8-infected SCID mice, we determined the viral titer in BAL fluid from the mice that had been challenged with 10 PFU, on day 7 and day 14 after infection, and also when mice had lost 25% of their initial body weight. The viral titer in the mice that had been treated with MAb 65 was significantly lower than that in the IgG2a control group at 7 dpi (*P* = 0.0378), 14 dpi (*P* = 0.0380), and at the end of the experiment (38 dpi, *P* = 0.0148) (Fig. 1C, experiment 1). Administration of MAb 65 even resulted in virus clearance in 6 out of 10 SCID mice in the first experiment. For the second experiment, lung virus loads were determined on 13 dpi (the day that the last mouse treated with control antibody had lost 25% of its initial body weight) and when the M2e MAb-treated mice had lost 25% of their initial body weight. Challenge of Mab 65-treated mice with 50 PFU of PR8 virus was associated with viral clearance in 2 out of 4 mice (Fig. 1C, experiment 2). The differences in viral titer between mice treated with M2e-specifc MAb 37, MAb 148, and control antibodies did not reach statistical significance (Fig. 1C).

### Whole-virus-genome deep sequence analysis.

We performed whole-PR8-genome deep sequence analysis using a workflow that we described before, with some adaptations in the influenza-specific RT-PCR protocol to allow amplification of viral RNA in BAL fluid samples with a low viral titer (38). The purified RT-PCR products of the two mouse experiments (experiment 1, *n* = 18; experiment 2, *n* = 25) were sequenced in two separate Illumina MiSeq runs. The minimum, maximum, and average coverage depth of PR8 virus genomic RNA isolated from individual mice were comparable within and between the 2 experiments, with the exception of mouse number 2 in the second experiment (IgG1 control-treated, ethical endpoint reached on day 10 after infection), which displayed an approximately 10-fold lower sequence coverage (see Table S1 in the supplemental material).

10.1128/mBio.00745-21.4 TABLE S1 Sequence coverage depth of PR8 virus sampled from bronchoalveolar lavage (BAL) fluid of treated severe combined immune-deficient (SCID) mice. Download Table S1, DOCX file, 0.02 MB. Copyright © 2021 Van den Hoecke et al. 2021 Van den Hoecke et al. https://creativecommons.org/licenses/by/4.0/ This content is distributed under the terms of the Creative Commons Attribution 4.0 International license.

High sequence diversity can be associated with increased fitness of the viral population. This has been shown, e.g., for poliovirus, where a mutant virus carrying a polymerase with a higher than natural fidelity expectedly exhibited a lower genetic diversity and turned out to be less fit in competition assays (39, 40). To address a possible change in sequence diversity of the PR8 virus in the M2e IgG-treated SCID mice, we determined the Shannon entropy for each of the eight genome segments and compared the entropy values for each genome segment between the different treatments. Within the same experiment, the Shannon entropy was similar in all samples for all conditions and all eight segments, suggesting that the anti-M2e MAb treatment did not alter the sequence diversity of PR8 (see Fig. S1 in the supplemental material). Next, we calculated the number of synonymous and nonsynonymous mutations that emerged over time after treatment. We found nucleotide differences compared with the sequence of the inoculum virus in all samples. The variants that were detected at a frequency above 10% are listed in Tables S2 to S7 in the supplemental material. Regardless of the treatment, nonsynonymous mutations at a frequency of 10% or higher were most frequently observed in HA and in one or more of the polymerase genes. In addition, nonsynonymous mutations in M2e were only observed in samples derived from mice that had been treated with MAb 37 or 65 (Tables S2 to S7).

10.1128/mBio.00745-21.1 FIG S1 Treatment of PR8-infected severe combined immune-deficient (SCID) mice with anti-M2e IgG monoclonal antibodies (MAbs) does not alter the virus genome sequence diversity. The standard Shannon entropy was calculated per segment and treatment (IgG1 or IgG2a isotype controls, anti-M2e MAbs [MAb 37, MAb 65, or MAb 148] or no treatment [noCPD, no compound]) after mapping the reads to the sample-specific majority rule consensus sequence (70). (A and B) Data obtained from experiments 1 and 2, respectively. Segment entropy levels among mice from the same condition were higher in the second experiment (all *P* values from an unpaired *t* test with Welch correction, <0.001). For these, the fraction of sites with nonzero entropy also differed (all *P* values, <0.001). Download FIG S1, PDF file, 0.1 MB. Copyright © 2021 Van den Hoecke et al. 2021 Van den Hoecke et al. https://creativecommons.org/licenses/by/4.0/ This content is distributed under the terms of the Creative Commons Attribution 4.0 International license.

10.1128/mBio.00745-21.5 TABLE S2 Variants detected above 10% in BAL fluid from untreated, PR8-infected SCID mice, isolated at 11 days postinfection (dpi). Download Table S2, DOCX file, 0.02 MB. Copyright © 2021 Van den Hoecke et al. 2021 Van den Hoecke et al. https://creativecommons.org/licenses/by/4.0/ This content is distributed under the terms of the Creative Commons Attribution 4.0 International license.

**FIG 2:**
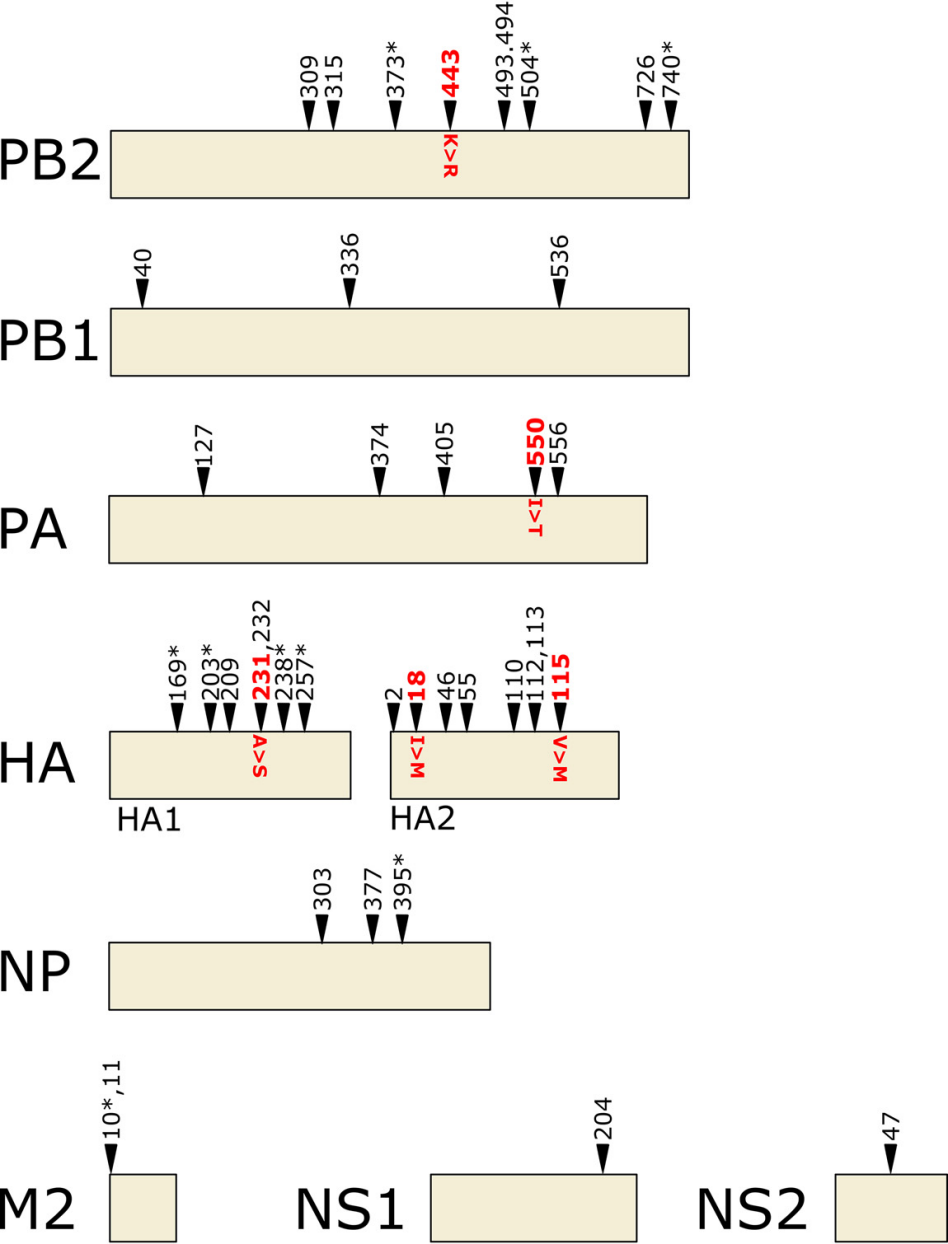
Mutations selected under M2e-specifc MAbs mapped to the primary structures of the viral proteins. Nonsynonymous mutations detected at a frequency greater than 50% are indicated schematically on the primary structural maps of the viral proteins. Mutations are indicated with arrowheads, with mutations that were detected in more than one sample marked with an asterisk. The following five prevalent nonsynonymous mutations found in an SCID mouse that had been treated with MAb 37 (EXP1; mouse no. 5; 33 dpi) and that were used to generated the rescued virus PR8-HPP are depicted in red: K443R in PB2 (62.7%), I550T in PA (98.09%), A231S in HA (96.32%), I361M (I18M in HA2) in HA (96.15%), and V458M (V115M in HA2) in HA (99.6%).

We mapped all of the sequence variants that were detected at frequencies above 50% in the anti-M2e MAb-treated samples to the primary structure of the viral proteins (Fig. 2). Most variants were present in the polymerase subunits PB2 and PA and in HA. No variants were detected in the NA segment with a frequency greater than 50%. Distance-based clustering analysis showed that the variants in the anti-M2e MAb- and control-treated groups clustered together (data not shown). The variants detected in the anti-M2e MAb-treated mice are thus not more similar to each other than to the variants that were present in the control-treated groups. In addition, no predictive polymorphism for either anti-M2e MAb-treated or control MAb-treated viral samples could be detected using logistic regression.

### M2e mutant viruses are selected in vivo with MAb 37 or MAb 65 but not with MAb 148.

**FIG 3:**
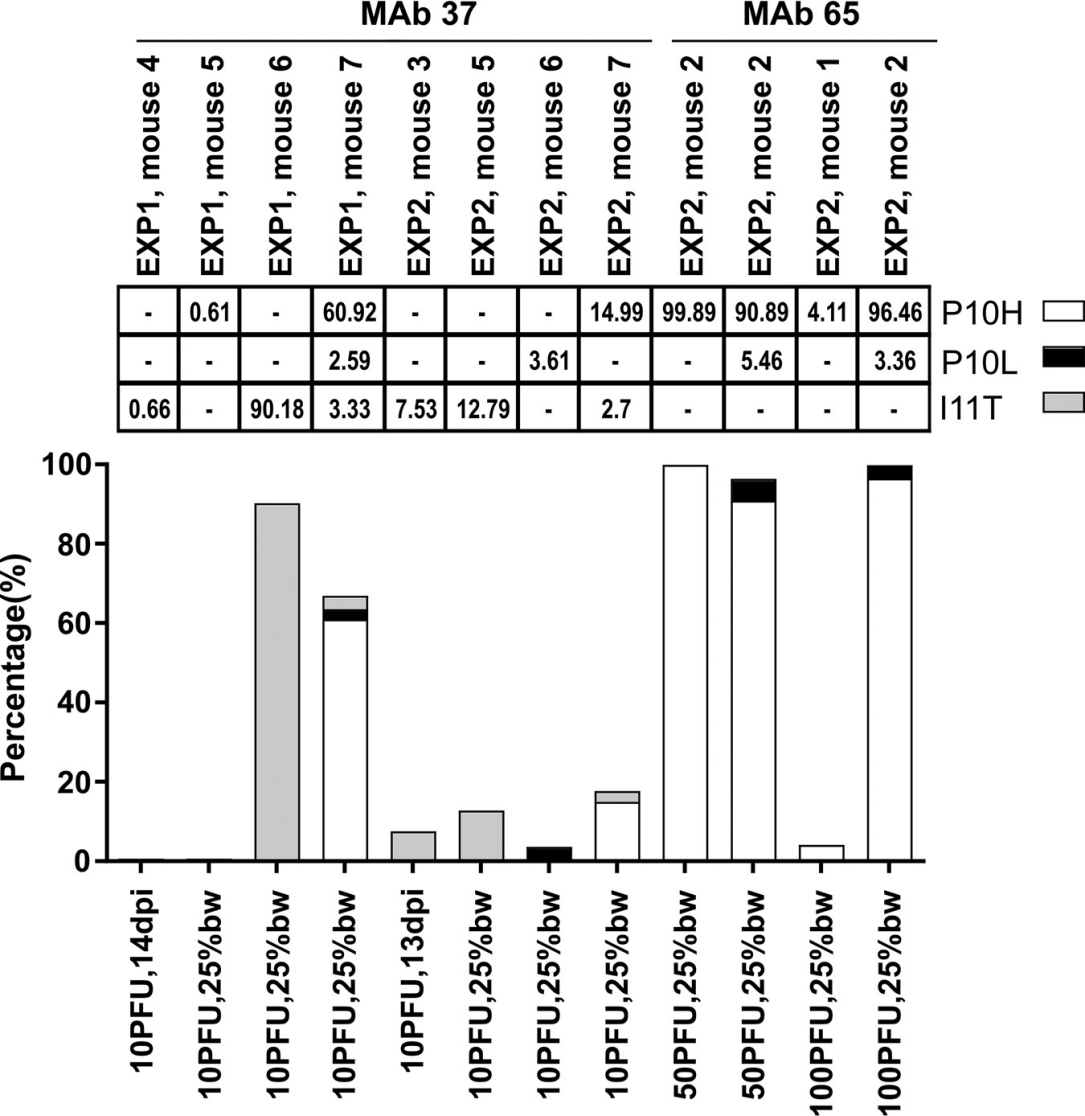
Changes in M2e associated with treatment with M2e-specific MAbs is limited to P10H, P10L, and I11T. Infected SCID mice were treated with M2e-specific MAb 37 or MAb 65. BAL fluid was isolated at the indicated time points (dpi), or when mice had lost 25% of their original body weight (25% bw), followed by influenza A virus genome-specific reverse transcription-PCR (RT-PCR) and Illumina MiSeq sequencing. The results for MAb 37 are from two independent experiments in which mice were infected with 10 PFU of PR8 virus. BAL fluid from MAb 65-treated mice was isolated when mice had lost at least 25% of their initial body weight after infection with either 50 or 100 PFU of PR8 virus. The presence of M2e variants was determined by Illumina MiSeq sequencing and is represented in the graph as percentages (%). Each bar represents a separate mouse, indicated by experiment number (EXP1 or 2) and mouse number. The frequency of each M2e variant (white, P10H; black, P10L; gray, I11T) is shown by the bars and in the grid above the graph.

The baseline sequence variation of M2e in the viral stock used to infect the mice and in all mice that received control treatment in the first mouse experiment was below the detection limit (38). Three types of variation were detected in M2e in virus samples derived from MAb 37- and MAb 65-treated mice, M2-P10H, M2-P10L, and M2-I11T (Fig. 3). The M2e mutations P10H, P10L, and I11T were detected at a frequency that ranged from 0.61% to 90.18% in 6 out of 10 mice treated with MAb 37 that reached the ethical endpoint (Fig. 3). In the second experiment, where MAb 65-treated mice had been infected with 50 or 100 PFU of PR8 virus, a change in the predicted M2e sequence was observed in all four mice that had lost 25% of their initial body weight (the ethical endpoint) with a frequency that ranged from 3.36% to 99.89% (Fig. 3). Interestingly, virus in the BAL fluid of all MAb 148-treated mice had a wild-type M2 sequence (Table S5). The variation in the M2e sequence was limited to nucleotide positions C762A (P10H) and C762T (P10L) for MAb 65. In MAb 37-treated mice, PR8 virus with a mutation at position T765C (I11T) was also observed. These three mutations are all synonymous in M1, hinting at a strong selection pressure to keep the M1 open reading frame intact.

10.1128/mBio.00745-21.8 TABLE S5 Variants detected above 10% in BAL fluid from MAb 148-treated mice infected with PR8, isolated at 13 dpi or when mice lost 25% of their initial body weight. Download Table S5, DOCX file, 0.03 MB. Copyright © 2021 Van den Hoecke et al. 2021 Van den Hoecke et al. https://creativecommons.org/licenses/by/4.0/ This content is distributed under the terms of the Creative Commons Attribution 4.0 International license.

**FIG 4:**
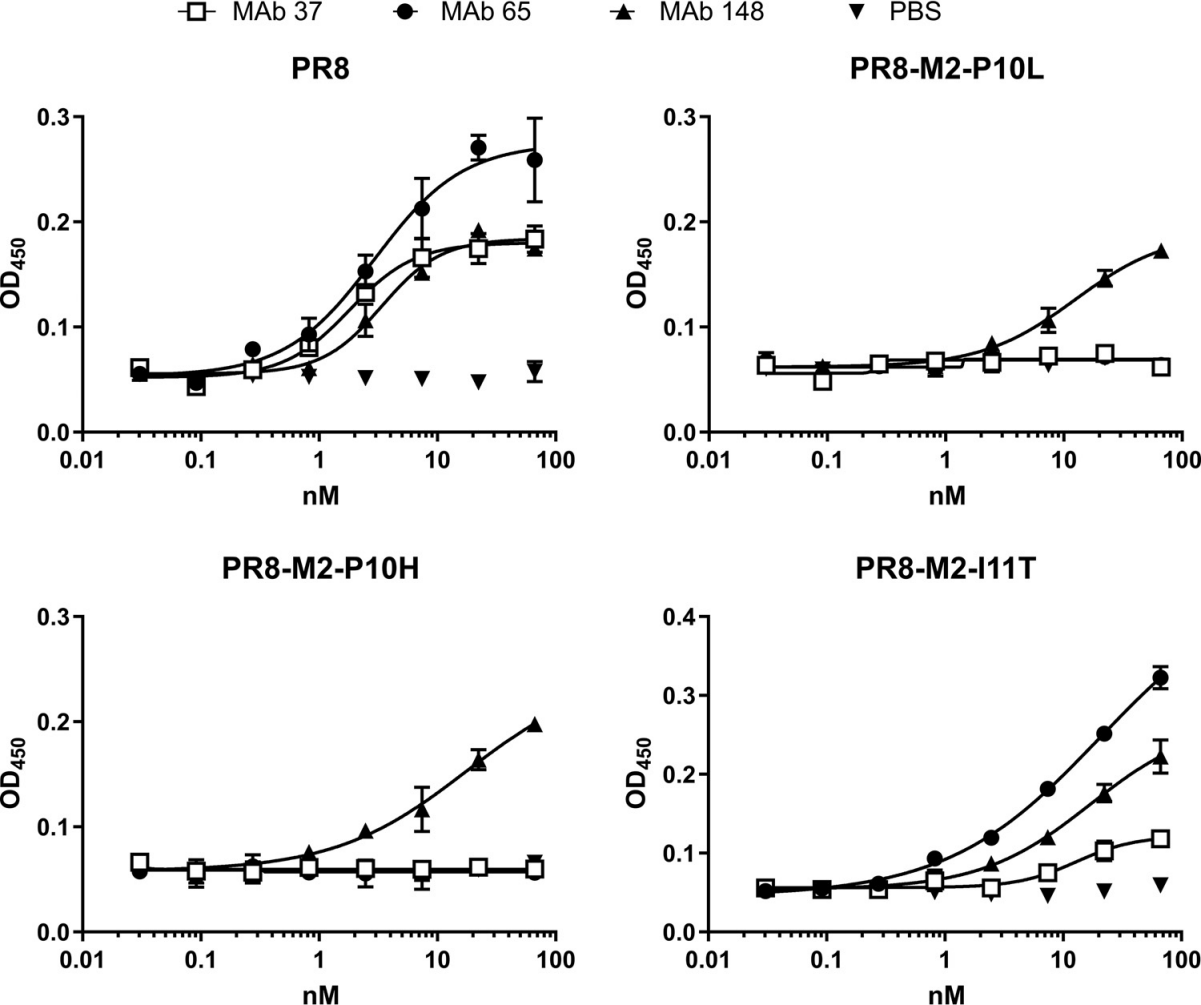
MAb 65 and MAb 37 fail to bind to M2e when a proline or histidine is present at position 10. MDCK cells were infected with A/Puerto Rico/8/34 (PR8) virus or a PR8 virus that carries a P10L, P10H, or I11T M2 mutation. Twenty-four hours later, the cells were incubated with a dilution series of MAb 37, MAb 65, MAb 148, or negative control (0.5% bovine serum albumin [BSA] in phosphate-buffered saline [PBS]) and fixed with 4% paraformaldehyde. Binding of the M2e-specific MAbs was revealed with horseradish peroxidase-conjugated sheep anti-mouse IgG as secondary antibody (Ab). OD_450_, optical density at 450 nm.

We previously reported that MAb 37 and MAb 65 recognize an overlapping epitope in M2e and bind to M2e, as present in PR8 virus, with similar affinity (30). However, the I11T mutation in M2e was only detected in MAb 37-treated mice, suggesting that position 11 of M2e is more critical for binding of MAb 37 than for binding of MAb 65. The ability of the M2e-specific MAbs to bind to the M2 mutant viruses was investigated using cell-based enzyme-limited immunosorbent assay (ELISA) with rescued recombinant PR8 viruses that carried either the P10L, P10H, or I11T mutation. Based on Sanger sequencing of the RT-PCR-amplified M2e region, these mutations were homogenously present in the virus stocks (unpublished result). MAb 148 bound to cells that had been infected with any of the four M2e variant PR8 viruses (Fig. 4). Cells that were infected with PR8-M2-P10L or PR8-M2-P10H viruses were not recognized by MAb 37 or MAb 65. In addition, MAb 37 bound only weakly to PR8-M2-I11T-infected cells, while MAb 65 efficiently recognized this M2e mutant (Fig. 4). This differential recognition of virus with M2-I11T by MAb 37 and MAb 65 is thus in line with the deep sequence analysis of the *in vivo* escape selection, in which M2-I11T mutant viruses were only observed in the MAb 37-treated mice.

To evaluate whether the acquired I11T mutation in M2 would suffice to escape MAb 37 treatment in our model, SCID mice were treated with MAb 37 or control IgG1 prior to and weekly after infection with 10 PFU of PR8 or PR8-M2-I11T virus. MAb 37 treatment protected against wild-type PR8 infection in this experiment (see Fig. S2 in the supplemental material. Two out of three control IgG-treated and two out of four MAb 37-treated SCID mice did not survive the PR8-M2-I11T challenge, suggesting that PR8 virus with a M2e-I11T mutation can escape MAb 37 treatment (Fig. S2).

10.1128/mBio.00745-21.2 FIG S2 MAb 37 does not protect SCID mice against PR8-M2-I11T challenge. One day before infection, SCID mice were intraperitoneally (i.p.) injected with 100 μg of MAb 37 (*n* = 8) or control IgG1 MAb (*n* = 7). MAb antibody (50-μg) injections were repeated at weekly intervals thereafter. Twenty-four hours after the first MAb injection, the mice were challenged with 10 PFU of PR8 (*n* = 8) or PR8-M2-I11T (*n* = 7) virus. Body weight and survival were monitored daily, and the experiment was terminated on day 24 after infection. Treatment with MAb 37 protected SCID mice against PR8 infection (**, *P* < 0.01 compared to IgG1 control; log-rank test). MAb 37 treatment protected partially (2/4 mice survived) against PR8-M2-I11T, whereas only 1 out of 3 IgG1 control-treated mice survived this challenge. Download FIG S2, PDF file, 0.03 MB. Copyright © 2021 Van den Hoecke et al. 2021 Van den Hoecke et al. https://creativecommons.org/licenses/by/4.0/ This content is distributed under the terms of the Creative Commons Attribution 4.0 International license.

### Prolonged *in vivo* replication selects for mutants with altered polymerase activity.

**FIG 5:**
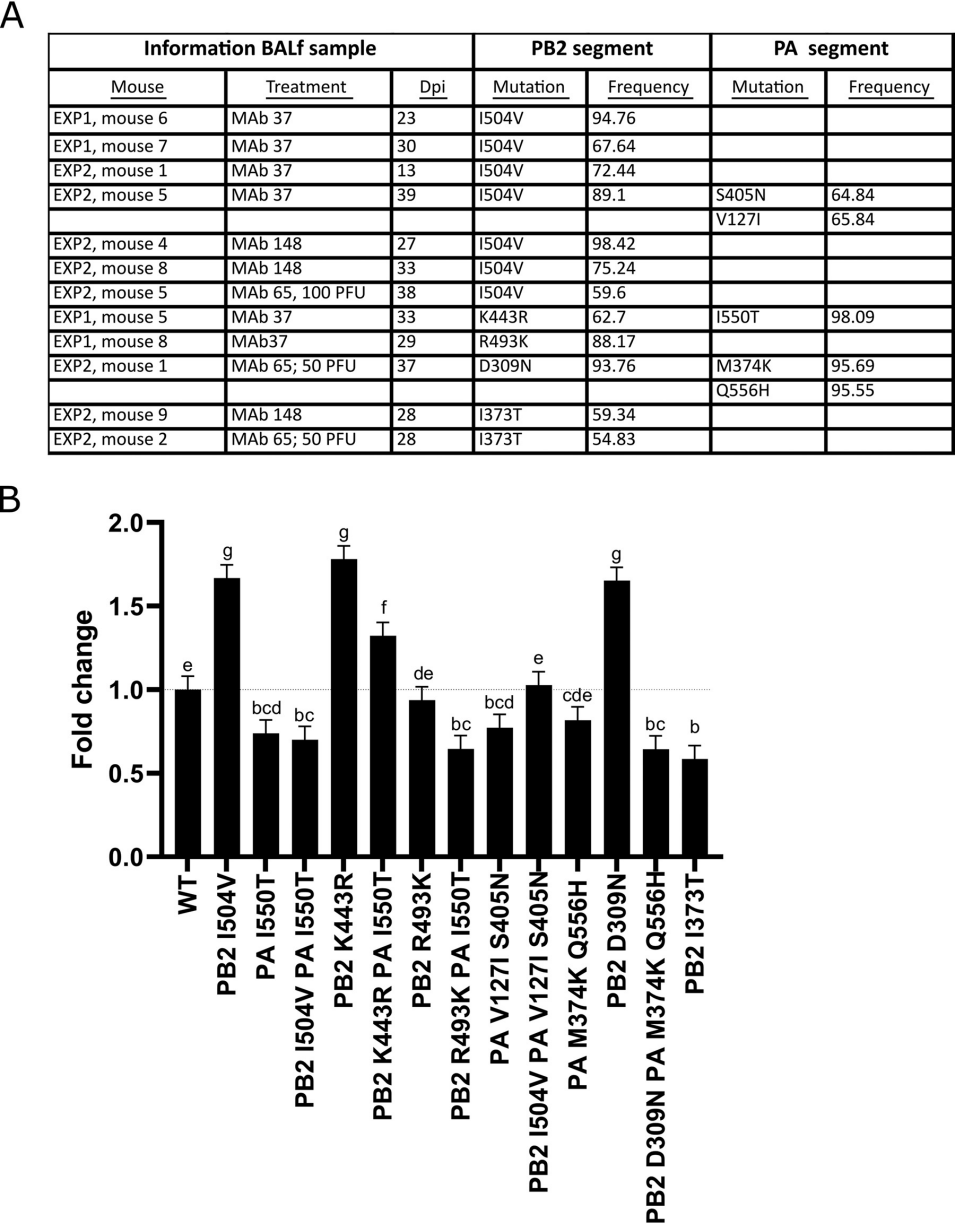
M2e-based immune selection pressure selects for viruses with PB2 and PA mutations that modulate polymerase activity. (A) Overview of the mutations in the PB2 and PA segment that occurred with a frequency of at least 50% in the next-generation sequencing (NGS) analysis and were tested in the minireplicon system. (B) HEK293T cells were transfected in triplicate with wild-type PB1 and NP combined with wild-type or mutant PB2 and wild-type PA or mutant PA expression plasmids (80 ng each), pHW-NSLuc (80 ng), and pRL-CMV (25 ng). The relative luciferase activity (firefly luciferase activity over *Renilla* luciferase activity) in the lysates was determined 24 h after transfection. Next, the wild-type condition was set to 1, and the fold change was calculated. Bars represent the averages from three independent experiments, each performed in triplicate. Error bars depict the effective standard error. Different superscript letters indicate significant difference (*P* < 0.05) compared with other conditions as assessed by analysis of variance (ANOVA) followed by a Fisher’s protected least significant difference procedure.

Our deep sequence analysis of the BAL fluid samples derived from anti-M2e MAb-treated mice revealed that a significant number of the detected variants were present in the viral polymerase subunits (Fig. 2 and Tables S2 to S7). We focused on PB2 and PA because several of the mutations that we identified in this study have been described before as being able to modulate viral polymerase activity and pathogenicity in mice (41–45). We evaluated a selection of nonsynonymous mutations in PB2 and PA that occurred at a frequency of at least 50% in the SCID BAL fluid samples in a minireplicon assay to assess their possible effects on viral polymerase activity (Fig. 5A). The PB2 mutation I504V, for example, was found at a frequency of 59.6% or greater in 7 BAL fluid samples derived from mice that had been treated with MAbs 37, 148, or 65, whereas PB1 and PA mutations were undetectable or were only present at low frequency (below 20%) in these samples (Tables S4 to S7). The mutations K443R (62.7%) in PB2 and I550T (98.09%) in PA occurred together in viruses that were isolated from a mouse that had been treated with MAb 37 (experiment 1, mouse 5, 33 dpi). In addition, the double mutation M374K and Q556H in PA occurred together with a D309N mutation in PB2 in viruses isolated from a MAb 65-treated mouse (experiment 2, mouse 1, 37 dpi). Viral RNA with two mutations in PA, V127I and S405N, combined with I504V in PB2 was detected in BAL fluid from a MAb 37-treated mouse (experiment 2, mouse 5, 39 dpi). We tested these mutations, as well as PB2-R493K, which emerged in another MAb 37-treated animal (experiment 1, mouse 8, 29 dpi), together with the well-documented I504V mutation in PB2 as a positive control, in a minireplicon assay on HEK cells (43). In addition, mutation PA I550T was included, since this mutation was identified in multiple escape virus populations in our study and it has been reported that PA I550L can enhance viral replication (43). Polymerase activity was significantly enhanced when PB2 D309N, PB2 I504V, or PB2 K443R were present in the otherwise wild-type polymerase complex (Fig. 5B). In contrast, polymerase activity was slightly reduced in the presence of PA I550T. Moreover, when combined with the polymerase activity-enhancing mutants PB2 R443K and PB2 I504V, PA I550T resulted in lower polymerase activity than that of the wild-type control (Fig. 5B). These minireplicon experiments support the conclusion that prolonged *in vivo* replication in SCID mice, which was accomplished by anti-M2e MAb treatment, selected for PR8 viruses with mutations in the PB2 and PA subunits that modulate the viral polymerase activity.

10.1128/mBio.00745-21.6 TABLE S3 Variants detected above 10% in BAL fluid sampled from control (A) IgG1- and (B) IgG2a-treated SCID mice. Download Table S3, DOCX file, 0.03 MB. Copyright © 2021 Van den Hoecke et al. 2021 Van den Hoecke et al. https://creativecommons.org/licenses/by/4.0/ This content is distributed under the terms of the Creative Commons Attribution 4.0 International license.

10.1128/mBio.00745-21.7 TABLE S4 Variants detected above 10% in BAL fluid from MAb 37-treated mice infected with PR8, isolated at 7, 13, or 14 dpi or when mice lost 25% of their initial body weight. Download Table S4, DOCX file, 0.04 MB. Copyright © 2021 Van den Hoecke et al. 2021 Van den Hoecke et al. https://creativecommons.org/licenses/by/4.0/ This content is distributed under the terms of the Creative Commons Attribution 4.0 International license.

### Viruses with delayed M2 expression evolve in SCID mice treated with MAb 37.

**FIG 6:**
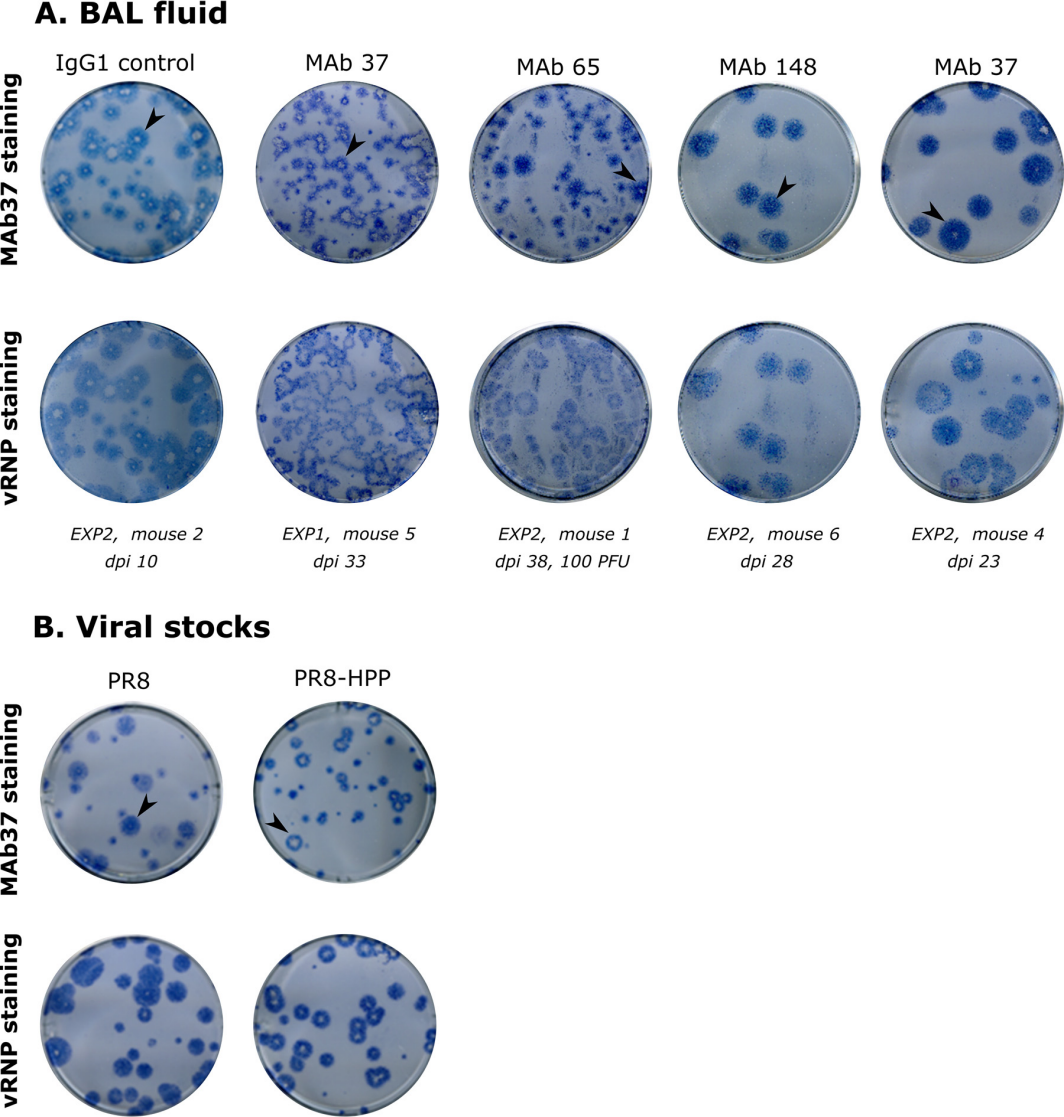
Plaques of virus isolated from a PR8-infected, MAb 37-treated mouse and a PR8-HPP-infected mouse show reduced M2e staining. MDCK cells were infected and fixed with 4% paraformaldehyde 2 days later. The plaques were revealed by immunostaining using anti-M2e specific MAb 37 or polyclonal anti-RNP serum (vRNP), followed by horseradish peroxidase-conjugated sheep anti-mouse IgG, respectively donkey anti-goat IgG, and TMB (3,3′,5,5′-tetramethylbenzidine) staining. (A) MDCK cells were infected with virus present in BAL fluid isolated from mice that had to be euthanized after infection with PR8 and different antibody treatments, as follows: IgG1 control MAb (EXP2; mouse 2; dpi 10), MAb 37 (EXP1; mouse 5; dpi 33), MAb 65 (EXP2; mouse 1; dpi 38; 100 PFU), MAb 148 (EXP2; mouse 6; dpi 28), or MAb 37 (EXP2; mouse 4; dpi 23). Plaques were first stained with MAb 37 (upper) and subsequently with polyclonal anti-RNP (lower). Sequence analysis revealed that viruses in these BAL samples had a wild-type M2e sequence. Five mutations were present above 50% in the viral population of one of the MAb 37-treated mice (EXP1; mouse 5; dpi 33): K443R in PB2, I550T and a silent mutation (G679A) in PA, A231S in HA1, and I18M in HA2. (B) MDCK cells were infected with PR8 (left) or PR8-HPP virus (right). The latter virus was generated by reverse genetics and carries the mutations K443R in PB2, I550T in PA, silent mutation G679A in HA, A231S in HA1, I18M in HA2, and V115M in HA2. Plaques were stained with MAb 37 (upper) or polyclonal anti-RNP (lower).

M2e-stained plaques (stained with MAb 37) derived from the BAL fluid of a SCID mouse that had been treated with MAb 37 displayed a smaller M2e immune-stained halo compared to the plaques of virus present in the BAL fluid of IgG1 control antibody-treated mice (Fig. 6A; compare IgG1 control [experiment 2, mouse 2, dpi 10] with MAb 37 [experiment 1, mouse 5; dpi 33]). In contrast, M2e-stained plaques of SCID mice that had been treated with MAb 65 (experiment 2, mouse 1, dpi 38, 100 PFU) or MAb 148 (experiment 2, mouse 6, dpi 28), as well as another MAb 37-treated SCID mouse (experiment 2, mouse 4, dpi 23), showed no clear difference in the size of the M2e halo compared to that in the IgG1 control sample (Fig. 6A).

Deep sequencing of the viral RNA present in the BAL fluid of the mouse that had been treated with MAb 37 (experiment 1, mouse 5, dpi 33) revealed a wild-type M2 sequence and five prevalent nonsynonymous mutations in other parts of the virus, namely, K443R in PB2 (62.7%), I550T in PA (98.09%), A231S in HA (96.32%), I361M (I18M in HA2) in HA (96.15%), and V458M (V115M in HA2) in HA (99.6%) (Table S4). Based on these frequencies, we calculated that close to 57% of the viral RNA present in this BAL fluid sample should have a combination of all five mutations. We thus rescued a PR8 virus with these mutations, which we named PR8-HPP. We noticed that, compared to wild-type PR8, plaques of PR8-HPP-infected MDCK cells had a smaller blue halo after anti-M2e MAb 37 staining (Fig. 6B). A possible explanation for this plaque phenotype could be that the timing of M2 expression in the cells infected with the rescued PR8-HPP virus was delayed.

**FIG 7:**
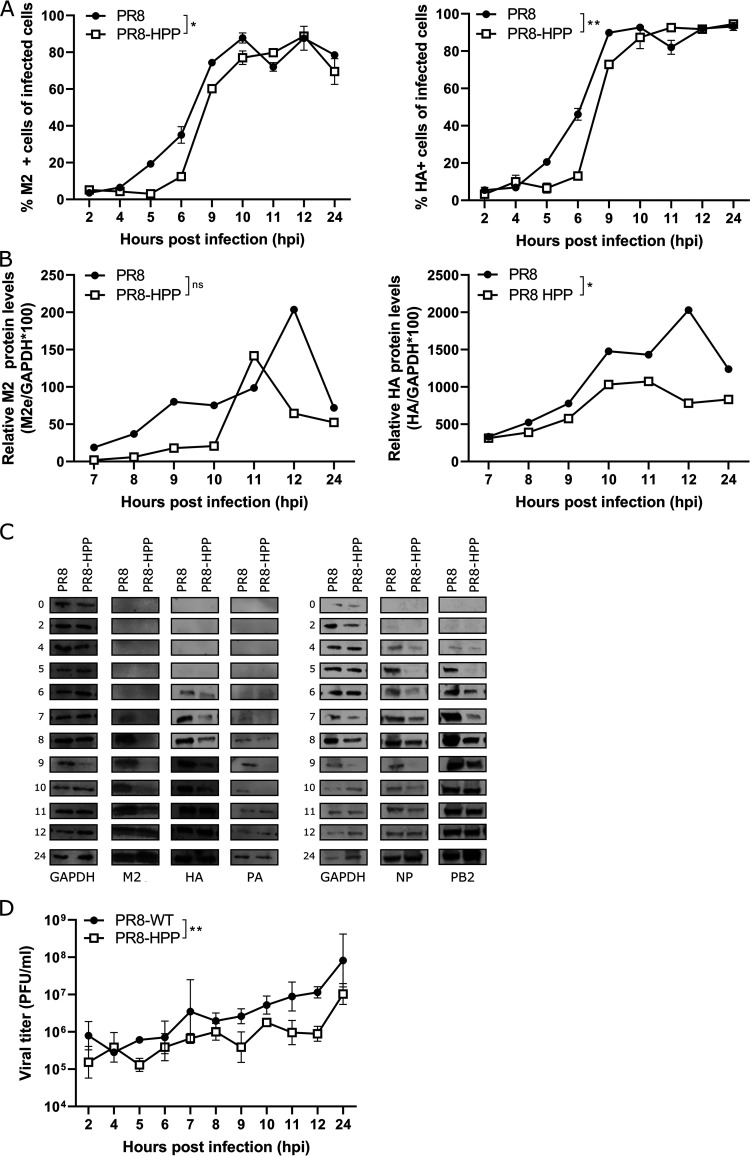
Viral protein expression, including M2, is delayed in PR8-HPP-infected cells. HEK293S cells were infected with a multiplicity of infection (MOI) of 1 of the wild-type PR8 or PR8-HPP virus and fixed with the fixation/permeabilization solution kit at the indicated time points. The samples were analyzed by flow cytometry (cells) (A) and Western blot analysis (cell lysates) (B and C). (A) HEK293S cells were stained with M2e-specific MAb 37 (left) or with a His-tagged HA-specific single-domain antibody followed by staining with anti His-tag antibody (right). All samples were analyzed on an LSR Fortessa 4 laser flow cytometer (BD). (B and C) Cell lysates were prepared at the indicated time points, and equal amounts of protein were separated based on size on an SDS-PAGE gel, followed by Western blotting to detect M2, HA, PA, NP, PB2, and GAPDH, which was used as a loading control. (B) Relative M2 (left) and HA (right) signal intensities in the Western blot were quantified and normalized to GAPDH (*n* = 1). Curves were compared using a two-way ANOVA test. (C) Representative Western blots showing protein levels at different time points (*n* = 1). (D) HEK293T cells were infected with PR8 or PR8-HPP virus (MOI = 1). The viral titer in supernatant at different time points was determined by viral plaque titration on MDCK cells. Curves were compared using a two-way ANOVA test (**, *P* < 0.05).

To explore this possibility, we performed *in vitro* virus growth kinetics experiments, in which the surface expression of M2 and HA, the latter of which carries three nonsynonymous mutations in the PR8-HPP mutant, was determined at different time points after infection of HEK293 suspension cells (HEK293S). Flow cytometry analysis of these samples showed that, compared to wild-type PR8, the cell surface expression of both M2 and HA after infection with PR8-HPP was delayed by approximately 1 h. At later time points, the cell surface expression of M2 and HA on PR8-HPP-infected cells increased to levels similar to those obtained for wild-type PR8-infected cells (Fig. 7A). In whole-cell lysates prepared at different time points after infection, M2 was detectable starting from 7 h postinfection (hpi) onwards for PR8 and only from 11 hpi onwards for PR8-HPP. HA becomes detectable for PR8 and PR8-HPP at 6 hpi, but the levels of PR8-HPP are lower until 11h after infection (Fig. 7B; see also Fig. S3A in the supplemental material). To investigate if this delayed or reduced protein expression was specific for M2 or HA, the expression of PA, PB2, and NP was evaluated in whole-cell lysates at different time points after infection (Fig. 7C; see Fig. S3B for full Western blots). PB2 expression was detectable for PR8 starting at 5 hpi and was slightly delayed in PR8-HPP-infected cells, in which expression was detected at 6 hpi at the earliest. A similar observation was made for NP expression. In addition, PA expression also appeared weaker in PR8-HPP-infected cells than in those infected with wild-type PR8 (Fig. 7C). Based on these results, we can conclude that in the context of PR8 virus, the combination of K443R in PB2, I550T in PA, and A231S, I361M, and V458M in HA is associated with delayed expression of M2, HA, NP, PA, and PB2. A delay in M2 expression compared to that in wild-type PR8 infection was not observed following infection with PR8-M2-I11T virus (Fig. S3C). For the other viral proteins, including HA, PA, and PB2, the expression profile was comparable between PR8-HPP- and PR8-M2-I11T-infected HEK293S cells (see Fig. S3C and Fig. S3D for full Western blots).

10.1128/mBio.00745-21.3 FIG S3 Viral protein expression, including that of M2, is delayed in PR8-HPP- and not in PR8-M2-I11T-infected cells. (A) Western blots showing M2 and HA protein levels after PR8 and PR8-HPP infection at different time points. HEK293S cells were infected with a multiplicity of infection (MOI) of 1 of the wild-type PR8 or PR8-HPP virus, and at the indicated time points cell lysates were made. The Western blot was stained for M2 and HA. GAPDH was used as a loading control. (B) Viral protein expression is delayed in PR8-HPP-infected cells compared to that in PR8-infected. Full Western blots used for Fig. 7C. HEK293S cells were infected with a MOI of 1 of the wild-type PR8 or PR8-HPP virus. Cell lysates were prepared on the indicated time points, and equal amounts of protein were separated based on an SDS-PAGE gel, followed by Western blotting to detect M2, HA, PA, NP, PB2, and GAPDH, which was used as a loading control. Specific protein bands are highlighted by a colored arrow specific for each protein. Five different blots were stained (blots A to E), and each blot was first visualized in the green channel (DyLight 800) followed by visualization in the red channel (DyLight 600). The protein marker can only be observed in the red channel. For the timepoints with two replicates on blots C and D, the sample with the asterisk was used for Fig. 7C. (C) Delayed M2 expression is specific for PR8-HPP-infected cells. HEK293S cells were infected with a MOI of 1 of the wild-type PR8, PR8-HPP, or PR8-M2-I11T virus. Cell lysates were prepared at the indicated time points, and equal amounts of protein were separated based on an SDS-PAGE gel, followed by Western blotting to detect M2, HA, PA, NP, PB2, and GAPDH, which was used as a loading control. Representative Western blot showing protein levels at different time points (*n* = 1). (D) Full Western blots used for panel C. HEK293S cells were infected with a MOI of 1 of the wild-type PR8, PR8-HPP, or PR8-M2-I11T virus. Cell lysates were prepared at the indicated time points, and equal amounts of protein were separated based on size on an SDS-PAGE gel, followed by Western blotting to detect M2, HA, PA, NP, PB2, and GAPDH, which was used as a loading control. Specific protein bands are highlighted by a colored arrow specific for each protein. Seven different blots were stained (blots A to G), and each blot was first visualized in the green channel (DyLight 800), followed by the red channel (DyLight 600). The protein marker can only be observed in the red channel. Download FIG S3, PDF file, 0.8 MB. Copyright © 2021 Van den Hoecke et al. 2021 Van den Hoecke et al. https://creativecommons.org/licenses/by/4.0/ This content is distributed under the terms of the Creative Commons Attribution 4.0 International license.

To investigate the possible impact of delayed expression of these important viral proteins on viral replication, viral titers in the supernatant were determined at different time points after infection of HEK293T cells. Viral titers in the cell supernatant increased over time for both PR8 and PR8-HPP. For PR8-HPP-infected cells, however, this increase was significantly lower than that in PR8-infected cells (Fig. 7D). In summary, the rescued PR8-HPP virus replicates at a lower rate than PR8 virus *in vitro*, which is associated with a reduced onset of expression of M2 and other viral gene products.

### PR8-HPP with delayed M2 expression is resistant to anti-M2e MAb 37 treatment in SCID mice.

**FIG 8:**
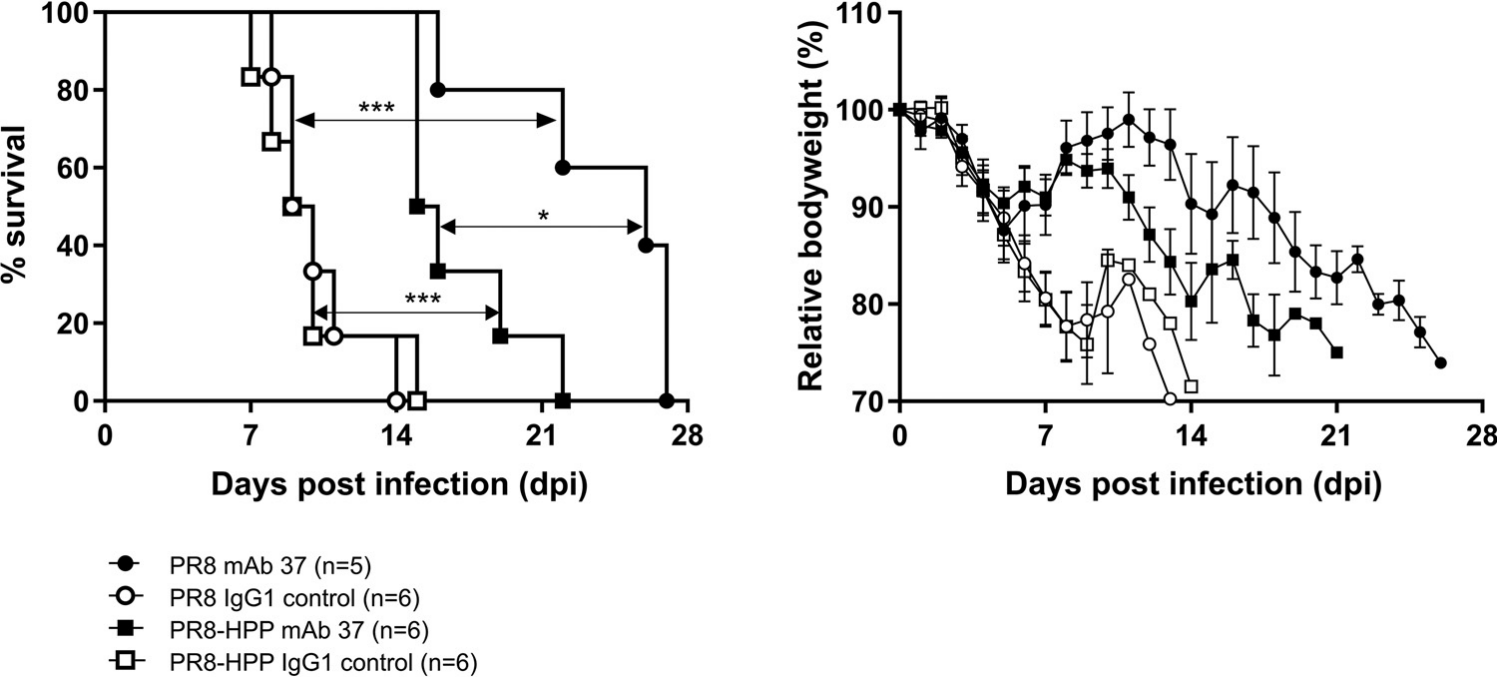
PR8-HPP, a virus with delayed M2 expression, is less sensitive than wild- type PR8 to anti-M2e IgG1 MAb treatment in the SCID model. One day before infection, SCID mice were i.p. injected with 100 μg of MAb 37 (*n* = 11) or IgG1 control MAb (*n* = 12), and MAb antibody (50 μg) injections were repeated at weekly intervals thereafter. At 24 h after the first MAb injection, the mice were challenged with 10 PFU of PR8 (*n* = 11) or PR8-HPP (*n* = 10) virus. Survival (left) and body weight (right) were monitored daily. In the body weight graph, data points represent averages and error bars standard deviations. Treatment with MAb 37 significantly prolonged survival compared to that of control-treated groups for both viruses (***, *P* < 0.001; Cox regression), although the effect of MAb 37 was more pronounced in the PR8-infected mice compared to that in the PR8-HPP-infected mice (*, *P* = 0.05; Cox regression).

To evaluate if the observed *in vitro* delayed viral protein expression could perturb *in vivo* protection by anti-M2e MAbs, we infected SCID mice with PR8 or the reconstituted PR8-HPP virus and treated these mice by intraperitoneal injection prior to and weekly after infection with anti-M2e MAb 37 or IgG1 isotype control antibody. The morbidity and mortality of the control IgG1-treated mice was comparable following infection with PR8 or PR8-HPP virus, indicating a similar pathogenicity of both viral strains in control IgG1-treated SCID mice (Fig. 8). Compared to the isotype control, treatment with MAb 37 significantly prolonged survival of PR8 and PR8-HPP virus-infected SCID mice (*P* < 0.001). However, survival of MAb 37-treated mice after infection with PR8-HPP was significantly reduced compared to that after PR8 virus infection (*P* < 0.05; Fig. 8). This demonstrates that a PR8 mutant virus with delayed expression of M2 (and other viral proteins) during viral infection is less sensitive to therapeutic intervention with an anti-M2e MAb. Delayed M2 expression may thus represent an alternative route for influenza viruses to escape from M2e immune pressure while maintaining a wild-type M2 sequence.

## DISCUSSION

We analyzed the genetic diversity of the influenza A virus PR8 under immune pressure evoked by 3 different M2e-specific MAbs in SCID mice at *in vivo* concentration levels that are similar to the M2e-specific serum antibody titers obtained after prime-boost vaccination of mice with M2e-MAP (40 to 80 μg/ml, based on M2e peptide ELISA, on the day of challenge [unpublished data]) (46). The absence of T and B cells in SCID mice also creates a well-controlled environment to study the viral diversity under prolonged anti-M2e IgG treatment. Although M2e-based immune protection is not neutralizing, and instead operates at the level of recognition and elimination of virus-infected cells, all three M2e-specific MAbs significantly prolonged the survival of PR8-infected mice compared to that in the control groups. In addition, in line with our previous results that showed that MAb 65 can bind to all activating FcγRs, this was translated into significantly better protection by MAb 65 compared to that by IgG1 MAb 37 in the SCID mouse model used here (30). MAb 65 treatment was even associated with virus clearance in SCID mice that had been challenged with a low dose of PR8 virus.

The deep *ex vivo* whole-virus-genome sequence analysis that we performed suggests that there are at least two different ways for the influenza A virus to evolve away from systemic monoclonal anti-M2e IgG immune pressure in the SCID model. The most obvious one is the outgrowth of a virus population with a mutation in the epitope that is recognized by the anti-M2e MAbs. In addition, we found that a virus can emerge with a wild-type M2 sequence and delayed M2 expression in infected cells. This delayed M2 expression compared with that of the parental virus, which was also apparent for the other viral gene products tested, was associated with acquired mutations in the polymerase subunits PB2 and PA and in HA.

Mutations in M2 were detected in mice that had been treated with MAb 37 and MAb 65, but not in viruses derived from mice that had been treated with MAb 148. The latter MAb recognizes the extremely conserved N-terminal part of M2 (33). In MAb 37- and MAb 65-treated mice, M2e diversity was only observed at positions 10 and 11 and was limited to P10L/H and I11T, which appeared either alone or in combination. These mutations are all silent for M1, suggesting that variation in M2e is limited by genetic restriction to M1. The appearance of these variants is also in line with the crystal structure of an Fab fragment of MAb 65 in complex with an M2e peptide that showed a critical role for E6, P10, I11, and W15 for MAb binding, although it was surprising that in none of the MAb 65-treated mice did a virus emerge with an I11 alteration in M2 like that readily found in MAb 37-treated mice (33). We have not yet determined the cocrystal structure of MAb 37 with an M2e peptide.

Active M2e-based vaccination strategies induce an oligoclonal anti-M2e response (47). We found that the M2e escape mutants can still be recognized by M2e immune serum that was raised with an M2e-virus like particle construct (unpublished data). In addition, Wolf et al. reported that mice that had been immunized with M2e-MAP, which carries four identical human consensus M2e peptides, were fully protected against challenge with PR8-M2 P10L or PR8-M2 P10H virus (48). Moreover, it has been stated that, when PR8-infected SCID mice were treated with a combination of MAbs that can bind to wild-type M2e, as well as P10H and P10L M2e variants, no viruses with an altered M2e sequence emerged (49). In addition, 11 consecutive passages of PR8 virus in M2e-vaccinated BALB/c mice did not result in selection of a M2e escape virus (49). From this, we can conclude that it may be unlikely that P10H/L or I11T escape mutants in M2e will emerge in a host that has been immunized with a M2e-based vaccine.

All viruses sampled from MAb 148-treated mice carried wild-type M2. This suggests that there is no or very little room for alterations in the sequence encoding the first nine amino acids of M2. In addition, MAb 148 could still bind to cells that had been infected with the P10H/L and I11T M2e escape variant viruses (Fig. 4). This is in line with the crystal structure of MAb 148 bound to M2e, where the deep and narrow binding pocket formed by the complementarity-determining regions of MAb 148 accommodates the N-terminal part of M2, with P10 and I11 emerging out of the paratope (34). Furthermore, treatment of experimentally infected individuals with a human M2e-specific MAb (TCN-032) did not result in viruses with an altered M2 sequence, as determined by 454 deep sequencing (5). These findings suggest that only a limited variability in M2e is tolerated, presumably as a consequence of the genetic constraint imposed by M1.

Treatment with anti-M2e MAbs, which significantly extended the time after infection before the ethical endpoint of the PR8-infected SCID mice was reached, not only created an environment in which anti-M2e-resistant PR8 viruses could emerge, but also provided a selective advantage for viruses that are even better adapted than the parental PR8 virus to the SCID mouse. In half of the BAL fluid samples of moribund MAb 37- and MAb 65-treated mice, and in all diseased MAb 148-treated mice, viruses were isolated with a wild-type M2 sequence but with nonsynonymous mutations in other gene segments, principally in the polymerase subunits and HA. Some of these mutations have been described previously. For example, the I504V mutation in PB2, which we identified in several of the challenged SCID mice, is associated with an increased replication efficiency and increased virus yield (42, 43, 50). Several of the mutations that were found in the current study (see Tables S2 to S7 in the supplemental material) have also been reported to be associated with influenza A virus mouse adaptation, e.g., D740N in PB2, M205I in PB1, and P199S in HA (41, 44, 51).

In all of the PR8-infected SCID mice, viruses that acquired mutations in the polymerases emerged at frequencies above 10%. The I504V mutation in PB2 was present at a frequency above 10% in virus isolated from half of the anti-M2e MAb-treated mice, and virus isolated from 20% of the mice carried a mutation at position 550 in PA. It has been reported that the I504V mutation in PB2 and I550L in PA increase the *in vitro* polymerase activity and that a virus that carries these mutations displays higher pathogenicity in immunocompetent mice (42, 43). In addition, we found several other mutations that have been linked to increased virulence in the polymerases at frequencies greater than 10%, e.g., D309N (52), R318K (41, 53), and R355K (45, 53, 54) in PB2 and V127I (41, 55) in PA. hvPR8, which contains, among others, the PB2 I504V and PA I550L mutations, is a PR8 variant that is virulent in Mx1-congenic mice, although it exhibits normal sensitivity to growth restriction by this protein (44). It has been proposed that the rapid replication of hvPR8 allows this virus to remain ahead of the interferon (IFN)-dependent restriction by mouse Mx1. We confirmed that the I504V mutation in PB2 enhances polymerase activity (Fig. 5). In addition, we also demonstrated that polymerase activity is enhanced when PB2 K443R or I504V are present. However, the PA I550T mutation, which emerged in some of the MAb 37- and MAb148-treated SCID mice, does not result in enhanced polymerase activity and even reduces the otherwise enhanced polymerase activity associated with PB2 K443R and PB2 I504V in the minireplicon system.

The fitness of influenza viruses can increase through mutations in the receptor binding domain (RBD) of HA. The crystal structure of HA from the PR8 virus revealed that it can bind equally well to α2,3- and α2,6-linked sialic acids (SA) (56). However, the mouse lung predominantly contains α2,3-linked SA (57). A D238G (D225G in H3 numbering) mutation was detected in several samples at a high frequency. This mutation is described as increasing binding to α2,3-linked SA (58, 59). In addition, the E169K (E156K in H3 numbering) mutation that emerged in several of our samples, is known to increase the receptor binding avidity of PR8 HA (60).

Our results reveal that reduced replication kinetics, which are associated with delayed M2 production, represent a possible escape route that does not involve a change in the targeted epitope. The reconstituted PR8-HPP virus (with PB2-K443R, PA-I550T, HA-A231S, HA-I361M, and HA-V458M mutations), however, displayed a general delay of viral protein expression *in vitro*. This suggests that early steps, such as entry or the delivery of the polyclonal anti-RNP sera (vRNP) to the nucleus, are affected in this virus since, eventually, the levels of M2 were found to be comparable to those of the wild-type virus. A shorter window between M2 expression, and hence M2e display, and new virion budding may enable virus release before the immune cells can attack M2-expressing cells with bound anti-M2e IgG antibody. Escape from M2e-specific immune pressure in a chronically infected, immunocompromised host is thus a complex process for which influenza viruses may use several strategies. Mutations in the M2e epitope can likely be prevented by including different M2e variants in a vaccine. Whether influenza A viruses with suppressed or delayed M2 expression could also emerge in an immunocompetent individual remains to be determined.

## MATERIALS AND METHODS

### Ethics statement.

All animal experiments described in this study were conducted according to the national (Belgian Law 14/08/1986 and 22/12/2003 and Belgian Royal Decree 06/04/2010) and European legislation on the protection of animals used for scientific purposes (EU directives 2010/63/EU and 86/609/EEC). All experiments on mice and all animal protocols were approved by the ethics committee of Ghent University (permit numbers EC2012-034 and EC2020-003). All efforts were made to avoid and ameliorate suffering of animals.

### Cell lines.

MDCK cells were cultured in Dulbecco’s modified Eagle medium (DMEM) supplemented with 10% fetal calf serum, nonessential amino acids, 2 mM l-glutamine, 100 U/ml penicillin, and 0.1 mg/ml streptomycin at 37°C in 5% CO_2_. HEK293T cells were cultured in the same medium, with the addition of 0.4 mM sodium pyruvate. HEK293S cells were cultured in a 1:1 mix of Freestyle-293 Expression medium (catalog no. 12338002; Life Technologies) and Ex-Cell 293 (catalog no. 14571C; Sigma).

### Antibodies used for *in vivo* treatment.

The M2e-specific antibodies MAb 65, MAb 37, and MAb 148 have been described previously (30, 33, 34). MAb 65 (IgG2a) and MAb 37 (IgG1) bind a similar epitope in M2e. MAb 148 binds the highly conserved N terminus of M2. Isotype control MAb directed against the ectodomain of NB of influenza B virus (IgG1) or the small hydrophobic protein of human respiratory syncytial virus (IgG2a) were used as irrelevant antigen-specific controls in passive transfer experiments. All antibodies were produced by conventional hybridoma technology and subsequently purified as described previously (30, 33).

### Infection, treatment, and analysis of mice.

C.B-17/IcrHan Hsd-Prkdc^scid^ (SCID) mice were purchased from Harlan. Mice were used at the age of 7 to 8 weeks and were housed in individually ventilated cages, in a temperature-controlled environment with 12-h light/dark cycles and food and water *ad libitum.* Influenza virus challenge experiments were performed in biosafety level 2+ facilities. To select for M2e escape mutants, mice were first injected i.p. with 100 μg (in a 200-μl volume) of MAb 37, MAb 65, or MAb 148, or isotype control MAbs. Twenty-four hours later, mice were anesthetized with a mixture of ketamine (10 mg/kg) and xylazine (60 mg/kg) and challenged by intranasal administration of 50 μl phosphate-buffered saline (PBS) containing 10 PFU of PR8 (50 or 100 PFU for the MAb 65 treatment in the second experiment). To evaluate if a virus that displays delayed M2 expression *in vitro* could change the *in vivo* protection by anti-M2e MAbs, SCID mice were injected i.p. with 100 μg of anti-M2e MAb 37 or IgG1 isotype control MAb. Twenty-four hours later, mice were challenged by intranasal administration of 10 PFU of PR8 or 10 PFU of the reconstituted PR8-HPP virus, as mentioned above. Next, the mice were treated with 50 μg of MAb 37 or IgG1 isotype control antibody i.p. weekly after infection. In all experiments, body weight and survival of mice were monitored daily after challenge. When mice lost more than 25% of their body weight, or on other days as otherwise mentioned, the mice were euthanized, and bronchoalveolar lavage (BAL) fluid was prepared by flushing the lungs two times with 600 μl of PBS. Cells in this BAL fluid were pelleted by centrifugation and the supernatants stored at −80°C.

### Cell-based ELISA.

MDCK cells were seeded in a 96-well plate at 25,000 cells per well. Sixteen hours later, the cells were infected with a multiplicity of infection (MOI) of 1 of PR8 virus or with the PR8-M2e mutant viruses (PR8-M2-P10H, PR8-M2-P10L, or PR8-M2-I11T). Twenty-four hours later, the cells were blocked with 0.5% bovine serum albumin (BSA) in PBS and incubated with MAb 37, MAb 65, or MAb 148, followed by fixation with 4% paraformaldehyde (PFA). After washing with 0.05% Tween 20 in PBS buffer, binding was detected using horseradish peroxidase (HRP)-conjugated sheep anti-mouse IgG antibodies (Abs).

### Plaque assay.

MDCK cells were seeded in complete DMEM in 12-well plates at 3 × 10^5^ cells per well. After 18 h, the cells were washed once with serum-free medium and incubated (in triplicate) with a 10-fold dilution series of the virus (made in serum-free cell culture medium containing 0.1% BSA) in 500 μl medium. After 1 h of incubation at 37°C, an overlay of 500 μl of 1.2% Avicel RC-591 (FMC BioPolymer) in serum-free medium with 4 μg/ml l-(tosylamido-2-phenyl) ethyl chloromethyl ketone (TPCK)-treated trypsin (Sigma) was added. After incubation at 37°C for 40 h, the overlay was removed, and the cells were fixed with 4% PFA and permeabilized with 20 mM glycine and 0.5% (vol/vol) Triton X-100. Plaques were stained with anti-M2e MAb 37 (final concentration, 0.5 μg/ml) followed by a secondary anti-mouse IgF HERP-linked antibody (GE Healthcare) or with polyclonal goat anti-influenza ribonucleoprotein (RN) (1/3,000, catalog no. NR-4282; Biodefense and Emerging Infections Resources Repository, NIAID, NIH) followed by secondary anti-goat IgG HRP-linked antibody (GE Healthcare). After washing, TrueBlue peroxidase substrate (KPL) was used to visualize the plaques.

### RT-PCR on BAL fluid samples.

The previously described RT-PCR protocol for full-genome influenza virus amplification was used to amplify the viral RNA present in BAL fluid samples (38). However, the lower viral titer in BAL fluid required further optimization of the RT-PCR conditions, and the following adaptations were made to the protocol: poly(A) RNA carrier (20 μg) was added to the lysis buffer used for RNA extraction; the DNase I digestion step was included during RNA isolation; the RNA elution buffer was preheated at 70°C; and the first 5 PCR cycles were performed at a lower annealing temperature (45°C instead of 72°C). In addition, two separate cDNA synthesis reactions were performed, using primers specific for the influenza A vRNAs, namely CommonUni12G (GCCGGAGCTCTGCAGATATCAGCGAAAGCAGG) and CommonUni12A (GCCGGAGCTC TGCAGATATCAGCAAAAGCAGG). Subsequently, all eight genomic segments were amplified in one PCR mixture of 100 μl using a mix containing 5 μl of CommonUni12G and CommonUni12A cDNA, 200 nM primer CommonUni13 (GCCGGAGCTCTGCAGATATCAGTA GAAACAAGG), and the Phusion high-fidelity polymerase (Thermo Scientific) (38, 61, 62). PCR products were purified using the High Pure PCR product purification kit (Roche) according to the manufacturer’s instructions, and the product was eluted in 50 μl sterile ultrapure water (preheated to 65°C).

### Illumina MiSeq library preparation and sequencing.

Aliquots of 300 ng (first experiment) or 150 ng (second experiment) of each RT-PCR sample were sheared with an M220 focused ultrasonicator (Covaris) set to obtain peak fragment lengths of 300 to 400 bp. Next, the NEBNext Ultra (experiment 1) or Ultra II (experiment 2, using dual indexing with NEBNext Multiplex Oligos for Illumina) DNA library preparation kit (New England Biolabs) was used to repair the ends and to add the Illumina MiSeq-compatible barcode adapters to 100 ng of fragmented DNA. The resulting fragments were then size selected using Agencourt AMPure XP bead sizing (Beckman Coulter). Afterwards, indexes were added in a limited-cycle PCR (10 cycles), followed by purification on Agencourt AMPure XP beads. Fragments were analyzed on a high-sensitivity DNA chip on a Bioanalyzer (Agilent Technologies). The multiplex sample was heat denatured for 2 min at 96°C before loading on the Illumina MiSeq chip. After the 2 × 250-bp Illumina MiSeq paired-end sequencing run, the data were base called, and reads with the same barcode were collected and assigned to a sample on the instrument, which generated Illumina FASTQ files (Phred +64 encoding).

### Data analysis.

The downstream data analyses were performed on the Illumina FASTQ files (Phred +64 encoding) using CLC Genomics Workbench (version 7.0.3) following the analysis pipeline described in Van den Hoecke et al., with quality trimming of the sequencing reads to a Phred score of 30 (38). For variant calling, the A-to-G variant introduced by the primer at position 24 in the HA, NP, NA, M, and NS segments was not considered during the influenza quasispecies variant analysis. In addition, the Val458Met variant in HA can be neglected, since this mutation was already present for 95% in the PR8 virus stock and is possibly the result of plaque purification (38).

### Generation and production of plasmids with escape mutations.

Reverse genetics plasmids for PR8 virus were kindly provided by Robert G. Webster (St. Jude Children's Research Hospital, Memphis, TN) (63). The mutations in pHW197-M, pHW193-PA, and pHW191-PB2 were introduced using QuikChange site-directed mutagenesis (Stratagene). The HA segment carrying the G679A (silent), G743T (Ala231Ser), A1135G (Ile361Met), and G1424A (Val458Met) mutations was introduced by Gibson cloning into the pHW2000 vector. The HA gene was first amplified by RT-PCR on vRNA using HAfw (GAAGTTGGGGGGGAGCAAAAGCAGGGGA) for cDNA synthesis (Transcriptor first-strand cDNA synthesis kit) and then PCR amplified with HAfv and HArv (CCGCCGGGTTATTAGTAGAAACAAGGgtg) for PCR. In parallel, overlapping ends were added to the pHW2000 vector by PCR, following the manufacturer’s protocol, using and flu-pHW-R (CCTGCTTTTGCTCCCCCCCAACTTC) and flu-pHW-F (CCTTGTTTCTACTAATAACCCGGCGG) as primers. DNA products were either purified from gel (for pHW2000) or from solution (for HA) using the High Pure PCR product purification kit (Roche). The Gibson reaction was performed according to the manufacturer’s instructions. All plasmids encoding either the wild-type or mutant PR8 genome segments were transformed and amplified in Escherichia coli DH5α. Plasmid DNA was isolated with a plasmid midikit (Qiagen) according to the manufacturer’s instructions. The resulting air-dried pellet was dissolved in 50 μl of sterile ultrapure water. The presence of the introduced mutations was confirmed by Sanger sequencing on a capillary sequencer (3730XL DNA Analyzer; Applied Biosystems).

### Generation of wild-type or mutant recombinant PR8 virus.

To generate recombinant wild-type PR8 virus, a mixture (1 μg of each plasmid) of pHW191-PB2, pHW192-PB1, pHW193-PA, pHW194-HA, pHW195-NP, pHW196-NA, pHW197-M, and pHW198-NS was transfected using calcium phosphate coprecipitation into a HEK293T-MDCK cell coculture in Opti-MEM (3 × 10^5^ HEK293T and 2 × 10^5^ MDCK cells in a 6-well plate). To generate mutant PR8 virus, the same setup was followed, but one or more of the wild-type pHW plasmids were replaced by the corresponding mutant plasmid. After 30 h, TPCK-treated trypsin (Sigma) was added to a final concentration of 2 μg/ml. After 72 h, the culture medium was collected, and the presence of virus was confirmed by hemagglutination of chicken red blood cells. Reverse genetics-generated PR8 and PR8 mutant viruses were plaque purified on MDCK cells as follows. Confluent MDCK cells in a 6-well plate were infected with a serial dilution series of virus. After 1 h, an overlay of low-melting-temperature agarose (type VII agarose, 1% final concentration; Sigma) in serum-free cell culture medium containing 4 μg/ml TPCK-treated trypsin (Sigma) was added. After 56 h, the cytopathic effect was checked, agar-overlaying viral plaques were selected with a pipette tip, and virus was allowed to diffuse from the agar for 24 h at 4°C in serum-free medium. Afterwards, virus derived from one plaque was amplified on MDCK cells in serum-free cell culture medium in the presence of 2 μg/ml TPCK-treated trypsin (Sigma). After 96 h, the culture medium was collected, and cell debris were removed by centrifugation for 10 min at 2,500 × *g* at 4°C, and the virus was pelleted from the supernatants by overnight centrifugation at 16,000 × *g* at 4°C. The pellet was dissolved in sterile 20% glycerol in PBS, aliquoted, and stored at −80°C. The infectious titer of the obtained virus stocks was determined by plaque assay on MDCK cells, on three different aliquots, each performed in triplicate.

### Determination of M2e sequence in viral stocks of PR8, PR8-M2-P10H, PR8-M2-P10L, and PR8-M2-I11T.

RNA was isolated using the High Pure RNA isolation kit (Roche) according to the manufacturer’s instructions. cDNA synthesis was performed using the influenza Uni12 (AGCAAAAGCAGG) primer and the Transcriptor first-strand cDNA synthesis kit (Roche) according to the manufacturer’s instructions for cDNA synthesis using gene-specific primers. The M segment was amplified using pHW-MPf (GAAGTTGGGGGGGAGCAAAAGCAGGTAG), pHW-MPr (CCGCCGGGTTATTAGTAGAAACAAGGTAG), Phusion polymerase (Thermo Scientific), and the following conditions: an initial denaturation step of 98°C for 30 min', followed by 5 cycles of 98°C for 10 min', 45°C for 30 min, and 72°C for 2 h and 30 cycles of 98°C for 10 min and 72°C for 150 min', and a final elongation step of 72°C for 7 h (64). Subsequently, the M segment was purified from 1% agarose gel (High Pure PCR product purification kit; Roche) and the presence of the introduced mutations confirmed by Sanger sequencing on a capillary sequencer (3730 XL DNA Analyzer; Applied Biosystems).

### Minireplicon assay.

HEK293T cells (seeded at 60,000 cells per well in 24-well plates) were transfected in triplicate with the expression plasmids pHW-PB1; pHW-PB2, pHW-PB2 I504V, pHW-PB2 K443R, pHW-PB2 R493K; pHW-PB2 I373T or pHW-PB2 D309N; pHW-PA, pHW-PA I550T, pHW-PA V127I, and S405N or pHW-PA M374K and Q556H; and pHW-NP (80 ng each); luciferase reporter pHW-NSLuc (80 ng); and pRL-CMV (25 ng) using polyethyleneimine as shown in Fig. 5B. For the negative-control samples, either pHW-PB2 or pHW-PA was substituted with empty vector pHW-2000. Cells were lysed 24 h later with passive lysis buffer (Promega). Luciferase activity was measured with the dual luciferase reporter assay system (E-1960; Promega) according to the manufacturer’s instructions, using a GloMax 96 microplate luminometer (Promega). The normalized luciferase activity was calculated as the ratio between the measured activities of firefly and *Renilla* luciferase (firefly/*Renilla* luciferase × 1,000).

### Flow cytometry analysis.

One day after seeding, 3 million HEK293S cells were infected with an MOI of 1 of either PR8 or PR8-HPP or left uninfected. At different time points after infection, cells were collected and thoroughly washed with PBS. One-third of the cells was used to make lysates for Western blot analysis, and the other two-thirds were further processed for fluorescence-activated cell sorting (FACS) analysis. After blocking with 1% BSA, the cells were stained with M2e-specific MAb 37 (10 μg/ml) and 10 μg/ml His-tagged HA-specific single-domain antibody (sdAb) described by Hufton et al. (65) in 0.5% BSA in PBS. Afterwards, the cells were washed (0.5% BSA in PBS) and treated with the fixation/permeabilization solution kit according to the manufacturer’s protocol (catalog no. 555028; BD). After fixation and permeabilization, cells were stained with polyclonal goat anti-influenza virus RNP (1/2,000, catalog no. NR-4282; Biodefense and Emerging Infections Resources Repository, NIAID, NIH) and monoclonal rabbit anti-His tag antibody (1/1,000; catalog no. PA1-983B; Thermo Scientific). Binding of primary antibodies was revealed with donkey anti-mouse IgG coupled to Alexa Fluor 594 (1/600; Invitrogen), donkey anti-rabbit IgG coupled to Alexa Fluor 488 (1/600; Invitrogen), and donkey anti-goat IgG coupled to Alexa Fluor 647 (1/600; Invitrogen), followed by analysis on an LSR Fortessa 4 laser flow cytometer (BD).

### Western blot analysis.

HEK293S cells were lysed in low-salt lysis buffer containing 50 mM Tris-HCl (pH 8.0), 150 mM NaCl, 1% Igepal (NP-40), and 5 mM EDTA at different times after infection. The samples were separated by SDS-PAGE (15%) and visualized by Western blotting with antibodies directed against M2e (MAb 37 or MAb 148, 1.3 μg/ml), RNP (polyclonal goat anti-RNP, 1/3,000, catalog no. NR-3133; Biodefense and Emerging Infections Resources Repository, NIAID, NIH), HA [polyclonal goat anti-influenza virus H1 (H0) hemagglutinin (HA) of A/Puerto Rico/8/1934 (H1N1), 1/1,000, catalog no. NR-3148; Biodefense and Emerging Infections Resources Repository, NIAID, NIH], PA (polyclonal rabbit, 1/1,000, catalog no. GTX125932; GeneTex), and PB2 (polyclonal rabbit, 1/1,000, catalog no. GTX125926; GeneTex), or GAPDH (polyclonal rabbit, 1/2,500, catalog no. ab9485; Abcam). For the detection, the following different DyLight-conjugated antibodies were used: goat IgG DyLight 680 conjugated (catalog no. 605-744-125; Rockland Immunochemicals, Inc.), goat anti-rabbit DyLight 800 conjugated (catalog no. 35571; Thermo Scientific), and goat anti-mouse DyLight 800 conjugated (catalog no. 35521; Thermo Scientific). Immunoreactive bands were detected using the Odyssey infrared imager (LI-COR Biosciences) and quantified using Image Studio Lite version 4.0.

### Shannon entropy.

To detect instances of convergent evolution, we compared the mother stock’s segment sequences with the newly assembled sample-specific majority rule consensus sequences (66). The *de novo* assembly was successful for 340/344 (98.8%) segments. Failure due to a wet-lab error was ruled out because a check with the Mosaik aligner revealed that numerous reads (>20,000) mapped to the corresponding segment of the mother stock for each of the four erroneous assemblies (67). All assemblies were visually inspected and, if required, manually edited in AliView (68). For optimal alignment accuracy, the newly generated segment assemblies were concatenated into sample-specific full genomes that served for the read mappings upon which a site-specific measure of nucleotide diversity (the standard Shannon entropy) was calculated (69). The corresponding segments of the closely related mother stock served to replace the four failed segment assemblies, and in the full genomes, the segments were separated from each other by strings of 50 nucleotides. Estimates of synonymous and nonsynonymous nucleotide diversity were obtained with SNPGenie (70). The entropy levels of the virus populations from mice that received the same treatment (i.e., IgG1 and MAb 37) in both sequencing experiments were contrasted with unpaired two-sided *t* tests, and for these populations, the proportion of sites with nonzero Shannon entropy levels was compared using Pearson’s chi-squared test with Yates’ continuity correction. R was used for the statistical analyses, and a *P* value of ≤0.05 was taken as the cutoff for statistical significance (71).

### Distance-based clustering.

To evaluate *in silico* whether lower-frequency variants can have a synergetic anti-M2e vaccine effect, we clustered the virus populations using several distance measures that are based on the presence and prevalence of single-nucleotide polymorphisms (SNPs) measured against the mother stock reference sequence. We used the following rules: (i) The distance is defined by the sum of unique polymorphic sites. In this setup, a site that is polymorphic in one population but homogenous in the other increases the distance between both populations by 1. (ii) As in rule i, but the type of polymorphism is also considered. In an example in which a site in population 1 has 7% A and 9% G as polymorphic content and the corresponding site in population 2 has 9% A and 0% G as nonreference nucleotides, the distance increases by 1. (iii) As in rule ii, but now the frequency of polymorphisms is also considered, and the distance increases with increasing prevalence differences. In the previous example, the distance increases by 11% (2 + 9). (iv) As in rule iii, but the distance is averaged over the number of characters considered. In the example, the distance increases by 5.5%. (v) Instead of the count, the distance is defined by the proportion of unique polymorphic sites. For example, population 1 has 10/40 polymorphic sites shared with population 2, and population 2 has 10/50 of its polymorphic sites in common. The distance between both is calculated as 30/40 + 40/50 = 1.55. (vi) As in rule v, but the average proportion is used as a proxy for the distance. In the previous example, the distance becomes 0.775. Distance calculations and hierarchical clustering visualizations were done in R for each of three SNP prevalence cutoffs (>5%, >25%, and >50%).

### Logistic regression.

We determined the association between the frequencies of SNPs and anti-M2e MAb treatment using a logistic regression. To increase the observation count for each outcome, the results of both sequencing experiments were combined with the sequence data obtained from a pilot SCID mice experiment in which the mice were challenged with 10 PFU of PR8 virus and were untreated (no compound). No-compound and IgG1- and IgG2a-treated populations were labeled as “controls,” and the other populations as “treatment.” Because using the same lower thresholds for polymorphic sites as above (e.g., >5%, >25%, and >50%) created a quasicomplete segregation, parameter estimates were obtained with penalized likelihood methods (72).

### Data availability.

The output sequencing reads were submitted to NCBI’s Sequence Read Archive under BioProject accession number PRJNA589271.

10.1128/mBio.00745-21.9 TABLE S6 Variants detected above 10% in BAL fluid isolated from MAb 65-treated mice infected with 50 PFU of PR8, when mice lost 25% of their initial body weight. Download Table S6, DOCX file, 0.02 MB. Copyright © 2021 Van den Hoecke et al. 2021 Van den Hoecke et al. https://creativecommons.org/licenses/by/4.0/ This content is distributed under the terms of the Creative Commons Attribution 4.0 International license.

10.1128/mBio.00745-21.10 TABLE S7 Variants detected above 10% in BAL fluid isolated from MAb 65-treated mice infected with 100 PFU of PR8, when mice lost 25% of their initial body weight. Download Table S7, DOCX file, 0.02 MB. Copyright © 2021 Van den Hoecke et al. 2021 Van den Hoecke et al. https://creativecommons.org/licenses/by/4.0/ This content is distributed under the terms of the Creative Commons Attribution 4.0 International license.
