# Supplementary material for: In Vivo Therapy with M2e-Specific IgG Selects for an Influenza A Virus Mutant with Delayed Matrix Protein 2 Expression
Source: mBio. 2021 Jul 13;12(4):e00745-21. doi: 10.1128/mBio.00745-21 (PMC8406285; doi:10.1128/mBio.00745-21)
Supplement: TABLE S1 [file mbio.00745-21-st001.docx]

Supplementary Table S1: Sequence coverage depth of PR8 virus sampled from BAL fluid of treated SCID mice.

Supplementary Table S1A: minimum, maximum and average sequencing coverage depth of PR8 virus genome after influenza-specific RT-PCR on RNA isolated from BAL fluid (BALf) of mice infected with 10 PFU of PR8, treated as indicated.

| 1^st^ mouse experiment | | | | | | |
| --- | --- | --- | --- | --- | --- | --- |
|  |  |  | **Coverage** | | |  |
| Treatment | **Mouse** | **Dpi** | **Minimum** | **Maximum** | **Average** | **SD (average)** |
| Untreated | 1 | 11 | 1619 | 57657 | 13005.40 | 12709.66 |
| Untreated | 2 | 11 | 2710 | 55245 | 13884.97 | 11506.39 |
| Untreated | 3 | 11 | 2222 | 64189 | 15135.92 | 14878.89 |
| Untreated | 4 | 11 | 2836 | 54232 | 13955.97 | 11021.56 |
| IgG1 control | 1 | 11 | 1359 | 88877 | 16647.49 | 16018.01 |
| IgG1 control | 2 | 15 | 1873 | 64429 | 15462.90 | 12603.65 |
| IgG1 control | 3 | 19 | 2056 | 59312 | 14943.63 | 11954.40 |
| IgG2a control | 1 | 12 | 2663 | 54214 | 14101.85 | 10369.01 |
| IgG2a control | 2 | 21 | 4781 | 51052 | 15286.96 | 9641.71 |
| IgG2a control | 3 | 12 | 4707 | 54629 | 15250.58 | 10161.71 |
| MAb 37 | 1 | 7 | 3423 | 63688 | 14835.94 | 11152.37 |
| MAb 37 | 2 | 7 | 3288 | 63826 | 14894.45 | 11863.82 |
| MAb 37 | 3 | 7 | 1795 | 81620 | 14582.97 | 13612.95 |
| MAb 37 | 4 | 14 | 5042 | 50911 | 14898.04 | 8684.95 |
| MAb 37 | 5 | 33 | 3801 | 51537 | 13466.80 | 11267.94 |
| MAb 37 | 6 | 23 | 5011 | 46412 | 15005.92 | 8403.85 |
| MAb 37 | 7 | 30 | 6450 | 45178 | 14918.95 | 8500.65 |
| MAb 37 | 8 | 29 | 5183 | 38011 | 14585.29 | 6299.66 |

SD = standard deviation

Supplementary Table S1B: minimum, maximum and average sequencing coverage depth of PR8 virus genome after influenza-specific RT-PCR on RNA isolated from BAL fluid of mice infected with 10 PFU (or 50 or 100 PFU as indicated) of PR8 treated as indicated.

| 2^nd^ mouse experiment | | | | | | |
| --- | --- | --- | --- | --- | --- | --- |
|  |  |  | **Coverage** | | |  |
| Treatment | **Mouse** | **Dpi** | **Minimum** | **Maximum** | **Average** | **SD (average)** |
| IgG1 control | 1 | 12 | 2782 | 38222 | 11108.53 | 8591.69 |
| IgG1 control | 2 | 10 | 287 | 4125 | 1369.99 | 838.77 |
| IgG1 control | 3 | 13 | 2255 | 35760 | 11113.39 | 8062.81 |
| MAb 37 | 1 | 13 | 4065 | 27855 | 10199.13 | 5189.11 |
| MAb 37 | 2 | 13 | 2749 | 33030 | 10877.47 | 5856.87 |
| MAb 37 | 3 | 13 | 2722 | 35154 | 10890.29 | 6177.49 |
| MAb 148 | 1 | 13 | 3453 | 38264 | 11670.28 | 6955.30 |
| MAb 148 | 2 | 13 | 3644 | 34769 | 10252.30 | 5950.33 |
| MAb 148 | 3 | 13 | 2229 | 32028 | 10294.68 | 5757.00 |
| MAb 37 | 4 | 23 | 3656 | 30083 | 12114.02 | 5725.89 |
| MAb 37 | 5 | 39 | 2880 | 28117 | 11167.71 | 5770.04 |
| MAb 37 | 6 | 20 | 3365 | 23340 | 8569.41 | 4455.49 |
| MAb 37 | 7 | 29 | 2804 | 28465 | 10403.31 | 5662.53 |
| MAb 37 | 8 | 23 | 4404 | 30516 | 11989.59 | 5585.34 |
| MAb 37 | 9 | 20 | 3672 | 27747 | 11047.84 | 5350.47 |
| MAb 148 | 4 | 27 | 3248 | 27188 | 10132.95 | 5965.50 |
| MAb 148 | 5 | 33 | 4499 | 31041 | 11259.96 | 6287.84 |
| MAb 148 | 6 | 28 | 3838 | 34936 | 13185.26 | 6993.80 |
| MAb 148 | 7 | 30 | 4723 | 36606 | 13174.48 | 7726.44 |
| MAb 148 | 8 | 33 | 2877 | 27126 | 10216.37 | 5347.08 |
| MAb 148 | 9 | 28 | 3000 | 26067 | 9699.04 | 5555.26 |
| MAb 65 (50 PFU) | 1 | 37 | 2754 | 61111 | 16909.90 | 13575.29 |
| MAb 65 (50 PFU) | 2 | 28 | 3162 | 27188 | 9459.08 | 5384.61 |
| MAb 65 (100 PFU) | 1 | 38 | 4504 | 34914 | 11287.38 | 6016.42 |
| MAb 65 (100 PFU) | 2 | 32 | 5149 | 49035 | 16497.54 | 9415.52 |

SD = standard deviation
