# Supplementary material for: In Vivo Therapy with M2e-Specific IgG Selects for an Influenza A Virus Mutant with Delayed Matrix Protein 2 Expression
Source: mBio. 2021 Jul 13;12(4):e00745-21. doi: 10.1128/mBio.00745-21 (PMC8406285; doi:10.1128/mBio.00745-21)
Supplement: FIG S1 [file mbio.00745-21-sf001.pdf]

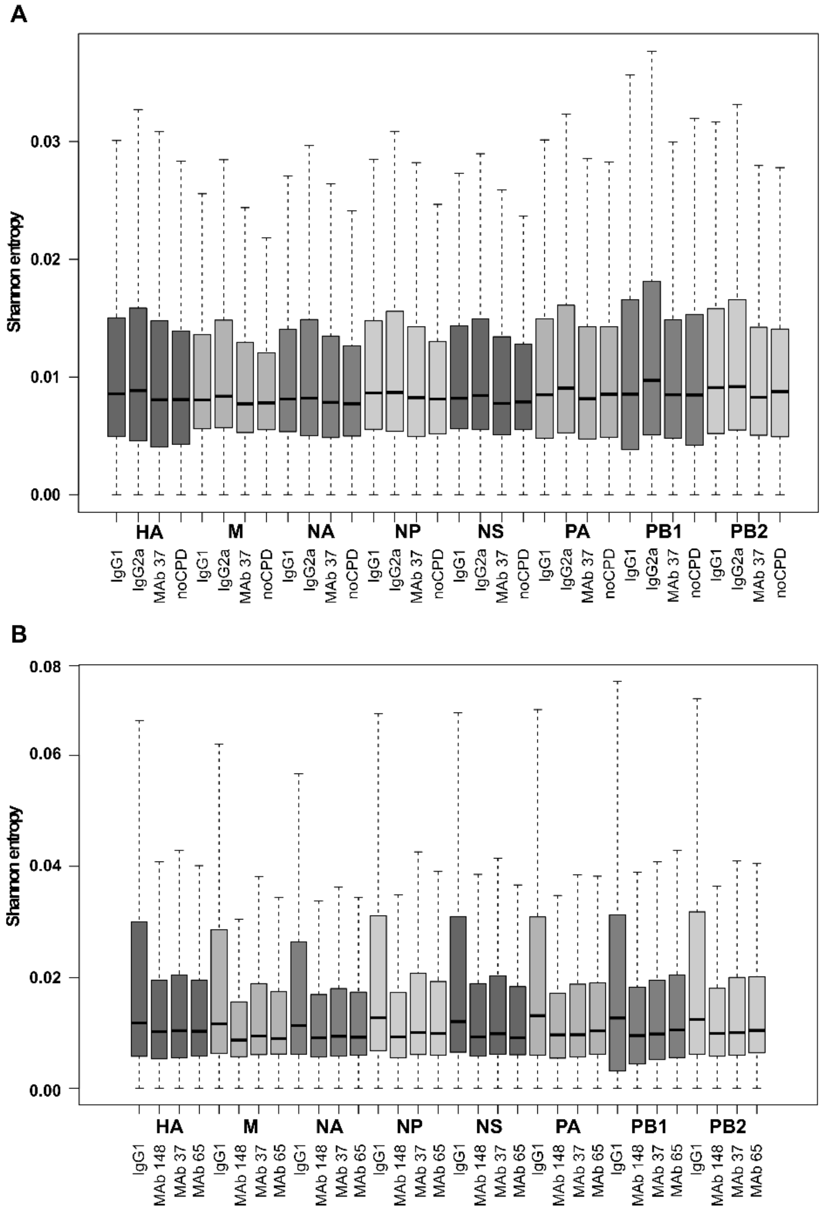

Supplementary Figure S1: Treatment of PR8-infected SCID mice with anti-M2e IgG mAbs does not alter the virus genome sequence diversity. The standard Shannon entropy was calculated per segment and treatment (IgG1 or IgG2a isotype controls, anti-M2e mAbs (MAb 37, MAb 65 or MAb 148) or no treatment (noCPD: no compound)) after mapping the reads to the sample-specific majority rule consensus sequence (1). Panel A and B represent data obtained from experiment 1 and 2, respectively. Segment entropy levels among mice from the same condition were higher in the second experiment (all p-values from an unpaired t-test with Welch correction < 0.001). For these, the fraction of sites with non-zero entropy also differed (all p-values < 0.001).

References

1. Nelson CW, Hughes AL. Within-host nucleotide diversity of virus populations: insights from next-generation sequencing. *Infect Genet Evol* 2015;30:1–7.
