# Supplementary material for: In Vivo Therapy with M2e-Specific IgG Selects for an Influenza A Virus Mutant with Delayed Matrix Protein 2 Expression
Source: mBio. 2021 Jul 13;12(4):e00745-21. doi: 10.1128/mBio.00745-21 (PMC8406285; doi:10.1128/mBio.00745-21)
Supplement: TABLE S2 [file mbio.00745-21-st002.docx]

Supplementary Table S2: Variants detected above 10% in BAL fluid from untreated, PR8-infected SCID mice, isolated at 11 dpi.

| Experiment |  | Dpi | Segment | Position | Frequency | Amino acid change |
| --- | --- | --- | --- | --- | --- | --- |
| 1^st^ | Mouse 1 | 11 | PB2 | 181 | 12.85 | PB2:p.Leu45Pro |
|  |  |  | PB2 | 184 | 15.61 | PB2:p.Arg46fs |
|  |  |  | PB2 | 340-354 | 10.22 | PB2:p.Trp98_Gly103delins* |
|  |  |  | PB1 | 74-87 | 26.05 | PB1:p.Leu10fs |
|  |  |  | HA | 648 | 11.66 | HA:p.Pro199His |
|  |  |  | HA | 749 | 13.79 | HA:p.Arg233Gly |
|  |  |  | HA | 765 | 24.44 | HA:p.Asp238Gly |
|  |  |  | HA | 1424 | 97.55 | HA:p.Val458Met |
|  |  |  | NP | 1249 | 32.99 | NP:p.Asn395Ser |
| 1^st^ | Mouse 2 | 11 | PB2 | 50 | 14.24 | PB2:p.Met1? |
|  |  |  | HA | 217 | 12.43 | Silent mutation |
|  |  |  | HA | 495 | 13.9 | HA:p.Val148Ala |
|  |  |  | HA | 765 | 12.16 | HA:p.Asp238Gly |
|  |  |  | HA | 770 | 18.02 | HA:p.Ala240Thr |
|  |  |  | HA | 847 | 12.83 | HA:p.Ile265Met |
|  |  |  | HA | 1424 | 86.94 | HA:p.Val458Met |
|  |  |  | M | 149 | 10.86 | M1:p.[Lys35Arg]^µ^ |
| 1^st^ | Mouse 3 | 11 | PB1 | 95-96 | 12.09 | PB1:p.Ala17fs^$^ |
|  |  |  | HA | 647 | 23.23 | HA:p.Pro199Phe |
|  |  |  | HA | 765 | 14.29 | HA:p.Asp238Gly |
|  |  |  | HA | 1424 | 99.94 | HA:p.Val458Met |
|  |  |  | M | 453 | 27.32 | Silent mutation |
| 1^st^ | Mouse 4 | 11 | PB2 | 2036 | 11.95 | Silent mutation |
|  |  |  | PA | 1265 | 10.09 | Silent mutation |
|  |  |  | HA | 497 | 11.06 | HA:p.Thr149Ala |
|  |  |  | HA | 749 | 14.38 | HA:p.Arg233Gly |
|  |  |  | HA | 765 | 21.37 | HA:p.Asp238Gly |
|  |  |  | HA | 1424 | 99.88 | HA:p.Val458Met |
|  |  |  | NA | 1240 | 12.89 | Silent mutation |

* = stop codon introduced with Trp98_Gly103delins

? = CDS will no longer be translated due to mutated translation initiating methionine

µ = mutation occurring in region linked to more than one coding sequence

$: fs = Frameshift mutation
