# Supplementary material for: In Vivo Therapy with M2e-Specific IgG Selects for an Influenza A Virus Mutant with Delayed Matrix Protein 2 Expression
Source: mBio. 2021 Jul 13;12(4):e00745-21. doi: 10.1128/mBio.00745-21 (PMC8406285; doi:10.1128/mBio.00745-21)
Supplement: TABLE S5 [file mbio.00745-21-st005.docx]

**Supplementary Table S5:** Variants detected above 10% in BAL fluid from MAb 148-treated mice infected with PR8, isolated at 13 dpi or when mice lost 25% of their initial body weight.

| Experiment |  | Dpi | Segment | Position | Frequency | Amino acid change |
| --- | --- | --- | --- | --- | --- | --- |
| 2^nd^ | Mouse 1 | 13 | PB2 | 1525 | 23.08 | PB2:p.Arg493Lys |
|  |  |  | HA | 689 | 66.48 | HA:p.Ala213Thr |
|  |  |  | HA | 1424 | 99.91 | HA:p.Val458Met |
|  |  |  | NP | 972 | 95.2 | NP:p.Pro303Ser |
|  |  |  | NA | 1180 | 21.04 | Silent mutation |
| 2^nd^ | Mouse 2 | 13 | HA | 1424 | 100 | HA:p.Val458Met |
|  |  |  | NP | 284 | 15.79 | Silent mutation |
|  |  |  | NS | 278 | 10.75 | NS1:p.Lys78Glu |
| 2^nd^ | Mouse 3 | 13 | PB2 | 972 | 14.51 | PB2:p.Asp309Asn |
|  |  |  | PB2 | 1000 | 46.72 | PB2:p.Arg318Lys |
|  |  |  | PB2 | 1110 | 10.69 | PB2:p.Arg355Gly |
|  |  |  | HA | 765 | 25.59 | HA:p.Asp238Gly |
|  |  |  | HA | 790 | 10.85 | Silent mutation |
|  |  |  | HA | 1424 | 100 | HA:p.Val458Met |
|  |  |  | NA | 916 | 11.23 | Silent mutation |
| 2^nd^ | Mouse 4 | 27 | PB2 | 1557 | 98.42 | PB2:p.Ile504Val |
|  |  |  | PA | 1534 | 19.07 | PA:p.[Lys497Arg];  PA-N155:p.[Lys343Arg];  PA-N182:p.[Lys316Arg] |
|  |  |  | HA | 765 | 84.22 | HA:p.Asp238Gly |
|  |  |  | HA | 1135 | 24.05 | HA:p.Ile361Met |
|  |  |  | HA | 1217 | 23.22 | HA:p.Asn389Asp |
|  |  |  | HA | 1424 | 99.91 | HA:p.Val458Met |
|  |  |  | NP | 1063 | 11.24 | NP:p.Cys333Phe |
|  |  |  | NA | 206 | 15.48 | NA:p.Tyr56His |
|  |  |  | NS | 657 | 92.63 | NS1:p.[Arg204Lys]; NS2:p.[Glu47Lys] |
| 2^nd^ | Mouse 5 | 33 | PB2 | 1557 | 25.88 | PB2:p.Ile504Val |
|  |  |  | PA | 981 | 40.03 | PA:p.[Thr313Ala];  PA-N155:p.[Thr159Ala];  PA-N182:p.[Thr132Ala] |
|  |  |  | PA | 1784 | 25.55 | PA:p.[Glu580Asp];  PA-N155:p.[Glu426Asp];  PA-N182:p.[Glu399Asp] |
|  |  |  | HA | 647 | 18.29 | HA:p.Pro199Ser |
|  |  |  | HA | 719 | 18.85 | HA:p.Asn223Tyr |
|  |  |  | HA | 823 | 15.2 | HA:p.Ile257Met |
|  |  |  | HA | 1424 | 99.79 | HA:p.Val458Met |
|  |  |  | NP | 542 | 12.71 | NP:p.Met159Ile |
|  |  |  | NA | 201 | 15.62 | NA:p.Ile54Asn |

**Supplementary Table S5 (continued):** Variants detected above 10% in BAL fluid from MAb 148-treated mice infected with PR8, isolated at 13 dpi or when mice lost 25% of their initial body weight.

| Experiment |  | Dpi | Segment | Position | Frequency | Amino acid change |
| --- | --- | --- | --- | --- | --- | --- |
| 2^nd^ | Mouse 6 | 28 | PB2 | 741 | 99.82 | Silent mutation |
|  |  |  | PB2 | 2223 | 53.35 | PB2:p.Ile726Val |
|  |  |  | PB1 | 1586 | 11.89 | Silent mutation |
|  |  |  | PA | 1052 | 10.46 | Silent mutation |
|  |  |  | PA | 1071 | 43.77 | PA:p.[Ala343Thr];  PA-N155:p.[Ala189Thr];  PA-N182:p.[Ala162Thr] |
|  |  |  | PA | 1293 | 12.33 | PA:p.[Leu417Met];  PA-N155:p.[Leu263Met];  PA-N182:p.[Leu236Met] |
|  |  |  | PA | 1711 | 10.34 | PA:p.[Gln556Arg];  PA-N155:p.[Gln402Arg];  PA-N182:p.[Gln375Arg] |
|  |  |  | HA | 775 | 10.43 | Silent mutation |
|  |  |  | HA | 1085 | 61.7 | HA:p.Leu345Ile |
|  |  |  | HA | 1419 | 38.43 | HA:p.Ser456Leu |
|  |  |  | NP | 212 | 99.97 | Silent mutation |
|  |  |  | NS | 151 | 12.18 | Silent mutation |
| 2^nd^ | Mouse 7 | 30 | PB2 | 1111 | 44.08 | PB2:p.Arg355Lys |
|  |  |  | PB1 | 2164 | 18.48 | PB1:p.[Arg707Lys];  PB1-N40:p.[Arg668Lys] |
|  |  |  | PA | 1692 | 28.84 | PA:p.[Ile550Val];  PA-N155:p.[Ile396Val];  PA-N182:p.[Ile369Val] |
|  |  |  | PA | 1887 | 24.51 | PA:p.[Lys615Glu];  PA-N155:p.[Lys461Glu];  PA-N182:p.[Lys434Glu] |
|  |  |  | HA | 611 | 44.59 | HA:p.Lys187Glu |
|  |  |  | HA | 765 | 85.81 | HA:p.Asp238Gly |
|  |  |  | HA | 1424 | 98.35 | HA:p.Val458Met |
|  |  |  | HA | 1598 | 37.88 | HA:p.Val516Ile |
|  |  |  | NP | 921 | 13.13 | NP:p.Ala286Ser |
|  |  |  | NP | 972 | 69.78 | NP:p.Pro303Ser |
|  |  |  | NP | 1420 | 18.4 | NP:p.Arg452Ile |
| 2^nd^ | Mouse 8 | 33 | PB2 | 782 | 10.39 | Silent mutation |
|  |  |  | PB2 | 1184 | 21.59 | Silent mutation |
|  |  |  | PB2 | 1557 | 75.24 | PB2:p.Ile504Val |
|  |  |  | PA | 51 | 49.46 | PA-X:p.[Asp3Asn]; PA:p.[Asp3Asn] |
|  |  |  | PA | 971 | 12.62 | PA:p.[Lys309Asn];  PA-N155:p.[Lys155Asn];  PA-N182:p.[Lys128Asn] |
|  |  |  | HA | 823 | 57.75 | HA:p.Ile257Met |
|  |  |  | HA | 856 | 15.1 | HA:p.Met268Ile |

**Supplementary Table S5 (continued):** Variants detected above 10% in BAL fluid from MAb 148-treated mice infected with PR8, isolated at 13 dpi or when mice lost 25% of their initial body weight.

| Experiment |  | Dpi | Segment | Position | Frequency | Amino acid change |
| --- | --- | --- | --- | --- | --- | --- |
| 2^nd^ | Mouse 8 | 33 | HA | 1135 | 10.93 | HA:p.Ile361Met |
|  |  |  | HA | 1415 | 15.23 | HA:p.Asp455Tyr |
|  |  |  | HA | 1417 | 50.34 | HA:p.Asp455Glu |
|  |  |  | HA | 1424 | 20.36 | HA:p.Val458Met |
|  |  |  | HA | 1537 | 49.52 | Silent mutation |
| 2^nd^ | Mouse 9 | 28 | PB2 | 1165 | 59.34 | PB2:p.Ile373Thr |
|  |  |  | PB1 | 659 | 13.03 | PB1:p.[Met205Ile];  PB1-N40:p.[Met166Ile] |
|  |  |  | PA | 1887 | 34.76 | PA:p.[Lys615Glu];  PA-N155:p.[Lys461Glu];  PA-N182:p.[Lys434Glu] |
|  |  |  | PA | 1933 | 26.24 | PA:p.[Glu630Gly];  PA-N155:p.[Glu476Gly];  PA-N182:p.[Glu449Gly] |
|  |  |  | HA | 557 | 77.46 | HA:p.Glu169Lys |
|  |  |  | HA | 765 | 16.91 | HA:p.Asp238Gly |
|  |  |  | HA | 1424 | 99.11 | HA:p.Val458Met |
|  |  |  | NA | 190 | 21.83 | NA:p.Asn50Lys |
|  |  |  | NA | 670 | 14.03 | Silent mutation |
|  |  |  | NS | 151 | 28.01 | Silent mutation |
