# Supplementary material for: In Vivo Therapy with M2e-Specific IgG Selects for an Influenza A Virus Mutant with Delayed Matrix Protein 2 Expression
Source: mBio. 2021 Jul 13;12(4):e00745-21. doi: 10.1128/mBio.00745-21 (PMC8406285; doi:10.1128/mBio.00745-21)
Supplement: FIG S2 [file mbio.00745-21-sf002.pdf]

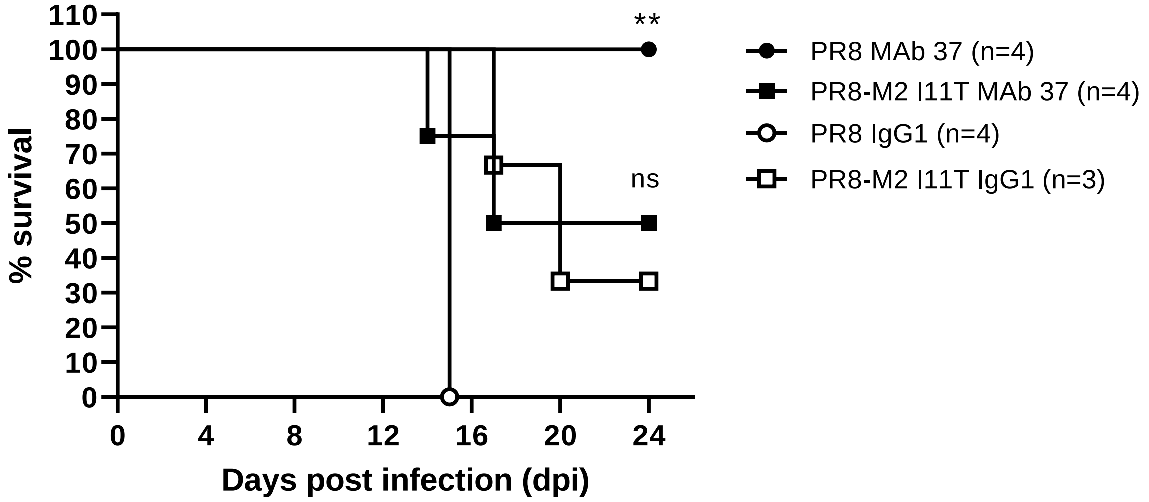

Supplementary Figure S2: MAb 37 does not protect SCID mice against PR8-M2 I11T challenge. One day before infection, SCID mice were i.p. injected with 100 µg of MAb 37 (n=8) or control IgG1 mAb (n=7). MAb antibody (50 µg) injections were repeated at weekly intervals thereafter. Twenty-four hours after the first mAb injection, the mice were challenged with 10 PFU of PR8 (n=8) or PR8-M2 I11T (n=7) virus. Body weight and survival were monitored daily and the experiment was terminated on day 24 after infection. Treatment with MAb 37 protected SCID mice against PR8 infection (** p< 0.01 compared to IgG1 control; LogRank test). MAb 37 treatment protected partially (2/4 mice survived) against PR8-M2 I11T whereas only 1 out 3 IgG1 control treated mice survived this challenge.
