# Supplementary material for: In Vivo Therapy with M2e-Specific IgG Selects for an Influenza A Virus Mutant with Delayed Matrix Protein 2 Expression
Source: mBio. 2021 Jul 13;12(4):e00745-21. doi: 10.1128/mBio.00745-21 (PMC8406285; doi:10.1128/mBio.00745-21)
Supplement: TABLE S3 [file mbio.00745-21-st003.docx]

Supplementary Table S3: Variants detected above 10% in BAL fluid sampled from control IgG1- and IgG2a-treated SCID mice.

Supplementary Table S3A: Variants detected above 10% in BAL fluid from control IgG1-treated mice infected with PR8, when mice lost 25% of their initial body weight.

| Experiment |  | Dpi | Segment | Position | Frequency | Amino acid change |
| --- | --- | --- | --- | --- | --- | --- |
| 1^st^ | Mouse 1 | 11 | PB1 | 675 | 11.99 | PB1:p.[Arg211Gly];  PB1-N40:p.[Arg172Gly] |
|  |  |  | HA | 493 | 18.87 | Silent mutation |
|  |  |  | HA | 765 | 26.48 | HA:p.Asp238Gly |
|  |  |  | HA | 770 | 21.87 | HA:p.Ala240Thr |
|  |  |  | HA | 847 | 10.54 | HA:p.Ile265Met |
|  |  |  | HA | 1424 | 75.32 | HA:p.Val458Met |
|  |  |  | NP | 1161 | 12.62 | NP:p.Ala366Ser |
|  |  |  | NP | 1249 | 16.42 | NP:p.Asn395Ser |
|  |  |  | M | 657 | 15.95 | Silent mutation |
| 1^st^ | Mouse 2 | 15 | PB2 | 2240 | 10.44 | Silent mutation |
|  |  |  | PB1 | 675 | 13.51 | PB1:p.[Arg211Gly];  PB1-N40:p.[Arg172Gly] |
|  |  |  | HA | 765 | 45.79 | HA:p.Asp238Gly |
|  |  |  | HA | 823 | 11.68 | HA:p.Ile257Met |
|  |  |  | HA | 1424 | 90.79 | HA:p.Val458Met |
|  |  |  | NP | 1249 | 15.08 | NP:p.Asn395Ser |
| 1^st^ | Mouse 3 | 19 | PB2 | 716 | 18.4 | Silent mutation |
|  |  |  | PB2 | 972 | 10.07 | PB2:p.Asp309Asn |
|  |  |  | PB1 | 675 | 13.58 | PB1:p.[Arg211Gly];  PB1-N40:p.[Arg172Gly] |
|  |  |  | PA | 188 | 49.4 | Silent mutation |
|  |  |  | PA | 2035 | 49.46 | PA:p.[Lys664Arg];  PA-N155:p.[Lys510Arg];  PA-N182:p.[Lys483Arg] |
|  |  |  | HA | 497 | 17.12 | HA:p.Thr149Ala |
|  |  |  | HA | 689 | 17.85 | HA:p.Ala213Thr |
|  |  |  | HA | 765 | 32.95 | HA:p.Asp238Gly |
|  |  |  | HA | 1424 | 99.94 | HA:p.Val458Met |
|  |  |  | NP | 434 | 45.62 | Silent mutation |

Supplementary Table S3A (continued): Variants detected above 10% in BAL fluid from control IgG1-treated mice infected with PR8, when mice lost 25% of their initial body weight.

| Experiment |  | Dpi | Segment | Position | Frequency | Amino acid change |
| --- | --- | --- | --- | --- | --- | --- |
| 2^nd^ | Mouse 1 | 12 | PB2 | 2009 | 11.8 | Silent mutation |
|  |  |  | HA | 1424 | 88.5 | HA:p.Val458Met |
|  |  |  | NP | 1187 | 19.98 | NP:p.Met374Ile |
| 2^nd^ | Mouse 2 | 10 | PB2 | 1000 | 10.6 | PB2:p.Arg318Lys |
|  |  |  | PA | 104-106 | 13.31 | PA-X:p.[Thr20_Met21delinsThr];  PA:p.[Thr20_Met21delinsThr] |
|  |  |  | HA | 500 | 10.43 | HA:p.Ala150Thr |
|  |  |  | HA | 765 | 14.49 | HA:p.Asp238Gly |
|  |  |  | HA | 770 | 10.61 | HA:p.Ala240Thr |
|  |  |  | HA | 1424 | 92.43 | HA:p.Val458Met |
|  |  |  | HA | 1528 | 25.22 | HA:p.Met492Ile |
| 2^nd^ | Mouse 3 | 13 | PB2 | 561 | 39.56 | PB2:p.Val172Met |
|  |  |  | PB2 | 1169 | 23.33 | Silent mutation |
|  |  |  | PB2 | 2158-2167 | 24.37 | PB2:p.Tyr704fs |
|  |  |  | PB1 | 79-91 | 10.99 | PB1:p.Val12fs |
|  |  |  | HA | 672 | 27.73 | HA:p.Leu207His |
|  |  |  | HA | 765 | 49.35 | HA:p.Asp238Gly |
|  |  |  | HA | 1424 | 99.98 | HA:p.Val458Met |
|  |  |  | NP | 1249 | 13.44 | NP:p.Asn395Ser |

Supplementary Table S3B: Variants detected above 10% in BALf isolated from control IgG2a-treated mice infected with PR8, when mice lost 25% of their initial body weight.

| Experiment |  | Dpi | Segment | Position | Frequency | Amino acid change |
| --- | --- | --- | --- | --- | --- | --- |
| 1^st^ | Mouse 1 | 12 | PB2 | 1355 | 16.14 | Silent mutation |
|  |  |  | PB1 | 1187 | 25.89 | Silent mutation |
|  |  |  | PB1 | 1331 | 12.2 | Silent mutation |
|  |  |  | HA | 442 | 34.4 | Silent mutation |
|  |  |  | HA | 749 | 10.64 | HA:p.Arg233Gly |
|  |  |  | HA | 765 | 12.15 | HA:p.Asp238Gly |
|  |  |  | HA | 847 | 36.14 | HA:p.Ile265Met |
|  |  |  | HA | 1424 | 98.44 | HA:p.Val458Met |
|  |  |  | HA | 1474 | 33.64 | Silent mutation |
|  |  |  | NA | 1251 | 11.29 | NA:p.Arg404Lys |
|  |  |  | M | 603 | 28.4 | Silent mutation |
| 1^st^ | Mouse 2 | 21 | PB2 | 416 | 18.01 | PB2:p.Glu123Asp |
|  |  |  | PB2 | 1557 | 17.21 | PB2:p.Ile504Val |
|  |  |  | PA | 102 | 14.61 | PA-X:p.[Thr20Ala]; PA:p.[Thr20Ala] |
|  |  |  | PA | 104 | 11.41 | Silent mutation |
|  |  |  | HA | 765 | 29.54 | HA:p.Asp238Gly |
|  |  |  | HA | 1424 | 99.88 | HA:p.Val458Met |
|  |  |  | NP | 1208 | 12.46 | Silent mutation |
| 1^st^ | Mouse 3 | 12 | HA | 765 | 22.62 | HA:p.Asp238Gly |
|  |  |  | HA | 1424 | 94.28 | HA:p.Val458Met |
