# Supplementary material for: In Vivo Therapy with M2e-Specific IgG Selects for an Influenza A Virus Mutant with Delayed Matrix Protein 2 Expression
Source: mBio. 2021 Jul 13;12(4):e00745-21. doi: 10.1128/mBio.00745-21 (PMC8406285; doi:10.1128/mBio.00745-21)
Supplement: TABLE S4 [file mbio.00745-21-st004.docx]

**Supplementary Table S4:** Variants detected above 10% in BAL fluid from MAb 37-treated mice infected with PR8, isolated at 7, 13 or 14 dpi or when mice lost 25% of their initial body weight.

| Experiment |  | Dpi | Segment | Position | Frequency | Amino acid change |
| --- | --- | --- | --- | --- | --- | --- |
| 1^st^ | Mouse 1 | 7 | HA | 1424 | 100 | HA:p.Val458Met |
|  |  |  | NP | 1249 | 37.76 | NP:p.Asn395Ser |
| 1^st^ | Mouse 2 | 7 | PB1 | 159-167 | 12.63 | PB1:p.[Thr39_Asp41del];  PB1-F2:p.[Thr7_Ile10delinsThr];  PB1-N40:p.[Met1?] |
|  |  |  | PB1 | 164 | 59.42 | PB1:p.[Met40fs]; PB1-F2:p.[Trp9fs];  PB1-N40:p.[Met1?] |
|  |  |  | PA | 136-139 | 12.19 | PA-X:p.[Glu31fs]; PA:p.[Glu31fs] |
|  |  |  | PA | 314-320 | 24.28 | PA-X:p.[Val90fs]; PA:p.[Val90fs] |
|  |  |  | HA | 1424 | 100 | HA:p.Val458Met |
|  |  |  | NP | 1249 | 73.26 | NP:p.Asn395Ser |
| 1^st^ | Mouse 3 | 7 | HA | 1424 | 100 | HA:p.Val458Met |
|  |  |  | HA | 1426 | 99.72 | Silent mutation |
|  |  |  | NP | 1249 | 99.93 | NP:p.Asn395Ser |
| 1^st^ | Mouse 4 | 14 | PB2 | 2218-2220 | 13.98 | PB2:p.Val724_Leu725delinsVal |
|  |  |  | PA | 166-175 | 14.55 | PA-X:p.[His41fs]; PA:p.[His41fs] |
|  |  |  | PA | 398 | 25.34 | Silent mutation |
|  |  |  | HA | 856 | 20.82 | HA:p.Met268Ile |
|  |  |  | HA | 1424 | 99.93 | HA:p.Val458Met |
| 1^st^ | Mouse 5 | 33 | PB2 | 1375 | 62.7 | PB2:p.Lys443Arg |
|  |  |  | PB1 | 737 | 13.03 | Silent mutation |
|  |  |  | PA | 1693 | 98.09 | PA:p.[Ile550Thr]; PA-N155:p.[Ile396Thr];  PA-N182:p.[Ile369Thr] |
|  |  |  | HA | 557 | 12.75 | HA:p.Glu169Lys |
|  |  |  | HA | 679 | 99.8 | Silent mutation |
|  |  |  | HA | 743 | 96.32 | HA:p.Ala231Ser |
|  |  |  | HA | 748 | 34.89 | HA:p.Glu232Asp |
|  |  |  | HA | 1135 | 96.15 | HA:p.Ile361Met |
|  |  |  | HA | 1424 | 99.6 | HA:p.Val458Met |
|  |  |  | NP | 267 | 22.87 | NP:p.Leu68Ile |
| 1^st^ | Mouse 6 | 23 | PB2 | 1557 | 94.76 | PB2:p.Ile504Val |
|  |  |  | PB1 | 1050 | 78.6 | PB1:p.[Val336Ile]; PB1-N40:p.[Val297Ile] |
|  |  |  | PA | 2006 | 10.64 | Silent mutation |
|  |  |  | HA | 191 | 18.29 | HA:p.Val47Ile |
|  |  |  | HA | 823 | 44.45 | HA:p.Ile257Met |
|  |  |  | HA | 1424 | 99.93 | HA:p.Val458Met |
|  |  |  | M | 765 | 90.26 | M2:p.[Ile11Thr] |

**Supplementary Table S4 (continued):** Variants detected above 10% in BAL fluid from MAb 37-treated mice infected with PR8, isolated at 7, 13 or 14 dpi or when mice lost 25% of their initial body weight.

| Experiment |  | Dpi | Segment | Position | Frequency | Amino acid change |
| --- | --- | --- | --- | --- | --- | --- |
| 1^st^ | Mouse 7 | 30 | PB2 | 796 | 12.21 | PB2:p.Val250Ala |
|  |  |  | PB2 | 1557 | 67.64 | PB2:p.Ile504Val |
|  |  |  | PB2 | 2102 | 13.29 | Silent mutation |
|  |  |  | PB1 | 686 | 18.26 | Silent mutation |
|  |  |  | PB1 | 1457 | 17.52 | Silent mutation |
|  |  |  | HA | 1244 | 11.82 | HA:p.Val398Ile |
|  |  |  | HA | 1424 | 99.81 | HA:p.Val458Met |
|  |  |  | HA | 1486 | 38.83 | Silent mutation |
|  |  |  | M | 762 | 60.95 | M2:p.[Pro10His] |
| 1^st^ | Mouse 8 | 29 | PB2 | 1525 | 88.17 | PB2:p.Arg493Lys |
|  |  |  | PB2 | 1557 | 12.88 | PB2:p.Ile504Val |
|  |  |  | PB1 | 925 | 12.03 | PB1:p.[Gln294Arg];  PB1-N40:p.[Gln255Arg] |
|  |  |  | HA | 738 | 10.49 | HA:p.Glu229Val |
|  |  |  | HA | 749 | 21.77 | HA:p.Arg233Gly |
|  |  |  | HA | 765 | 36.33 | HA:p.Asp238Gly |
|  |  |  | HA | 1039 | 25.66 | Silent mutation |
|  |  |  | HA | 1424 | 99.72 | HA:p.Val458Met |
|  |  |  | HA | 1426 | 11.09 | Silent mutation |
| 2^nd^ | Mouse 1 | 13 | PB2 | 165-182 | 13.72 | PB2:p.Glu40_Leu45del |
|  |  |  | PB2 | 238 | 12.97 | PB2:p.Thr64fs |
|  |  |  | PB2 | 1557 | 72.44 | PB2:p.Ile504Val |
|  |  |  | PB1 | 1364 | 18.6 | Silent mutation |
|  |  |  | HA | 933 | 10.75 | HA:p.Lys294Arg |
|  |  |  | HA | 1424 | 99.93 | HA:p.Val458Met |
|  |  |  | HA | 1426 | 11.31 | Silent mutation |
|  |  |  | HA | 1619 | 99.88 | HA:p.Ser523Pro |
| 2^nd^ | Mouse 2 | 13 | PB1 | 794 | 35.47 | Silent mutation |
|  |  |  | HA | 611 | 20.64 | HA:p.Lys187Glu |
|  |  |  | HA | 1424 | 99.73 | HA:p.Val458Met |
|  |  |  | NP | 1324 | 24.49 | NP:p.Phe420Cys |
|  |  |  | M | 1024 | 10.87 | Silent mutation |

**Supplementary Table S4 (continued):** Variants detected above 10% in BAL fluid from MAb 37-treated mice infected with PR8, isolated at 7, 13 or 14 dpi or when mice lost 25% of their initial body weight.

| Experiment |  | Dpi | Segment | Position | Frequency | Amino acid change |
| --- | --- | --- | --- | --- | --- | --- |
| 2^nd^ | Mouse 3 | 13 | PB2 | 1111 | 22.57 | PB2:p.Arg355Lys |
|  |  |  | PB1 | 1650 | 99.87 | PB1:p.[Asn536Asp];  PB1-N40:p.[Asn497Asp] |
|  |  |  | HA | 740 | 20.69 | HA:p.Ile230Val |
|  |  |  | HA | 1424 | 99.91 | HA:p.Val458Met |
| 2^nd^ | Mouse 4 | 23 | PB2 | 1557 | 15.7 | PB2:p.Ile504Val |
|  |  |  | PB2 | 2265 | 72.61 | PB2:p.Asp740Asn |
|  |  |  | HA | 765 | 99.96 | HA:p.Asp238Gly |
|  |  |  | HA | 1424 | 99.91 | HA:p.Val458Met |
|  |  |  | NP | 212 | 96.29 | Silent mutation |
| 2^nd^ | Mouse 5 | 39 | PB2 | 1557 | 89.1 | PB2:p.Ile504Val |
|  |  |  | PA | 413 | 70.91 | Silent mutation |
|  |  |  | PA | 423 | 65.84 | PA-X:p.[Val127Ile]; PA:p.[Val127Ile] |
|  |  |  | PA | 1258 | 64.84 | PA:p.[Ser405Asn];  PA-N155:p.[Ser251Asn];  PA-N182:p.[Ser224Asn] |
|  |  |  | HA | 427 | 85.24 | Silent mutation |
|  |  |  | HA | 660 | 87.75 | HA:p.Glu203Gly |
|  |  |  | HA | 747 | 10.4 | HA:p.Glu232Gly |
|  |  |  | HA | 1424 | 98.25 | HA:p.Val458Met |
|  |  |  | NA | 415 | 90.07 | Silent mutation |
|  |  |  | NA | 492 | 27.7 | NA:p.Ala151Val |
|  |  |  | M | 765 | 12.86 | M2:p.[Ile11Thr] |
| 2^nd^ | Mouse 6 | 20 | PB2 | 992 | 51.5 | PB2:p.Met315Ile |
|  |  |  | PB1 | 490 | 11.15 | PB1:p.[Val149Ala];  PB1-N40:p.[Val110Ala] |
|  |  |  | PA | 51 | 33.07 | PA-X:p.[Asp3Asn]; PA:p.[Asp3Asn] |
|  |  |  | PA | 1421 | 42.22 | PA:p.[Ile459Met]; PA-N155:p.[Ile305Met];  PA-N182:p.[Ile278Met] |
|  |  |  | HA | 765 | 70.77 | HA:p.Asp238Gly |
|  |  |  | HA | 1424 | 100 | HA:p.Val458Met |
|  |  |  | NP | 925 | 12.71 | NP:p.Ser287Asn |
| 2^nd^ | Mouse 7 | 29 | PB2 | 864 | 23.52 | PB2:p.Ser273Thr |
|  |  |  | PB2 | 1132 | 22.55 | PB2:p.Glu362Gly |
|  |  |  | PB2 | 2120 | 62.96 | Silent mutation |
|  |  |  | PB2 | 2216-2217 | 31.18 | PB2:p.Asn723fs |
|  |  |  | PB1 | 1434 | 11.42 | PB1:p.[Asp464Asn];  PB1-N40:p.[Asp425Asn] |
|  |  |  | PB1 | 2214 | 23.74 | PB1:p.[Ile724Val];  PB1-N40:p.[Ile685Val] |

**Supplementary table S4 (continued):** Variants detected above 10% in BAL fluid from MAb 37-treated mice infected with PR8, isolated at 7, 13 or 14 dpi or when mice lost 25% of their initial body weight.

| Experiment |  | Dpi | Segment | Position | Frequency | Amino acid change |
| --- | --- | --- | --- | --- | --- | --- |
| 2^nd^ | Mouse 7 | 29 | PA | 753 | 47.09 | PA-X:p.[Glu237Lys]; PA:p.[Glu237Lys];  PA-N155:p.[Glu83Lys];  PA-N182:p.[Glu56Lys] |
|  |  |  | HA | 359 | 13.66 | HA:p.Glu103Lys |
|  |  |  | HA | 1217 | 24.86 | HA:p.Asn389Asp |
|  |  |  | HA | 1409 | 26.78 | HA:p.Phe453Leu |
|  |  |  | HA | 1424 | 72.17 | HA:p.Val458Met |
|  |  |  | NA | 820 | 26.12 | Silent mutation |
|  |  |  | M | 155 | 42.29 | M1:p.[Thr37Ile] |
|  |  |  | M | 762 | 15.25 | M2:p.[Pro10His] |
|  |  |  | NS | 592 | 15.31 | NS2:p.[Ser25Leu] |
| 2^nd^ | Mouse 8 | 23 | PB2 | 1091 | 13.68 | Silent mutation |
|  |  |  | PB2 | 1557 | 33.28 | PB2:p.Ile504Val |
|  |  |  | PB2 | 1570 | 12.85 | PB2:p.Arg508Gln |
|  |  |  | PB1 | 67 | 11.77 | PB1:p.Leu8Pro |
|  |  |  | PB1 | 1244 | 10.55 | Silent mutation |
|  |  |  | PA | 1887 | 17.54 | PA:p.[Lys615Glu];  PA-N155:p.[Lys461Glu];  PA-N182:p.[Lys434Glu] |
|  |  |  | HA | 748 | 99.96 | HA:p.Glu232Asp |
|  |  |  | HA | 1424 | 99.95 | HA:p.Val458Met |
|  |  |  | NP | 110 | 13.76 |  |
| 2^nd^ | Mouse 9 | 20 | PB1 | 2214 | 15.49 | PB1:p.[Ile724Val];  PB1-N40:p.[Ile685Val] |
|  |  |  | PA | 1292 | 10.49 | PA:p.[Glu416Asp];  PA-N155:p.[Glu262Asp];  PA-N182:p.[Glu235Asp] |
|  |  |  | PA | 1661 | 11.37 | Silent mutation |
|  |  |  | PA | 1692 | 19.66 | PA:p.[Ile550Val];  PA-N155:p.[Ile396Val];  PA-N182:p.[Ile369Val] |
|  |  |  | PA | 1694 | 11.45 | PA:p.[Ile550Met];  PA-N155:p.[Ile396Met];  PA-N182:p.[Ile396Met] |
|  |  |  | HA | 678 | 89.88 | HA:p.Gln209Arg |
|  |  |  | HA | 1424 | 99.79 | HA:p.Val458Met |
