# Supplementary figures and images for: In Vivo Therapy with M2e-Specific IgG Selects for an Influenza A Virus Mutant with Delayed Matrix Protein 2 Expression
Source: mBio. 2021 Jul 13;12(4):e00745-21. doi: 10.1128/mBio.00745-21 (PMC8406285; doi:10.1128/mBio.00745-21)

A

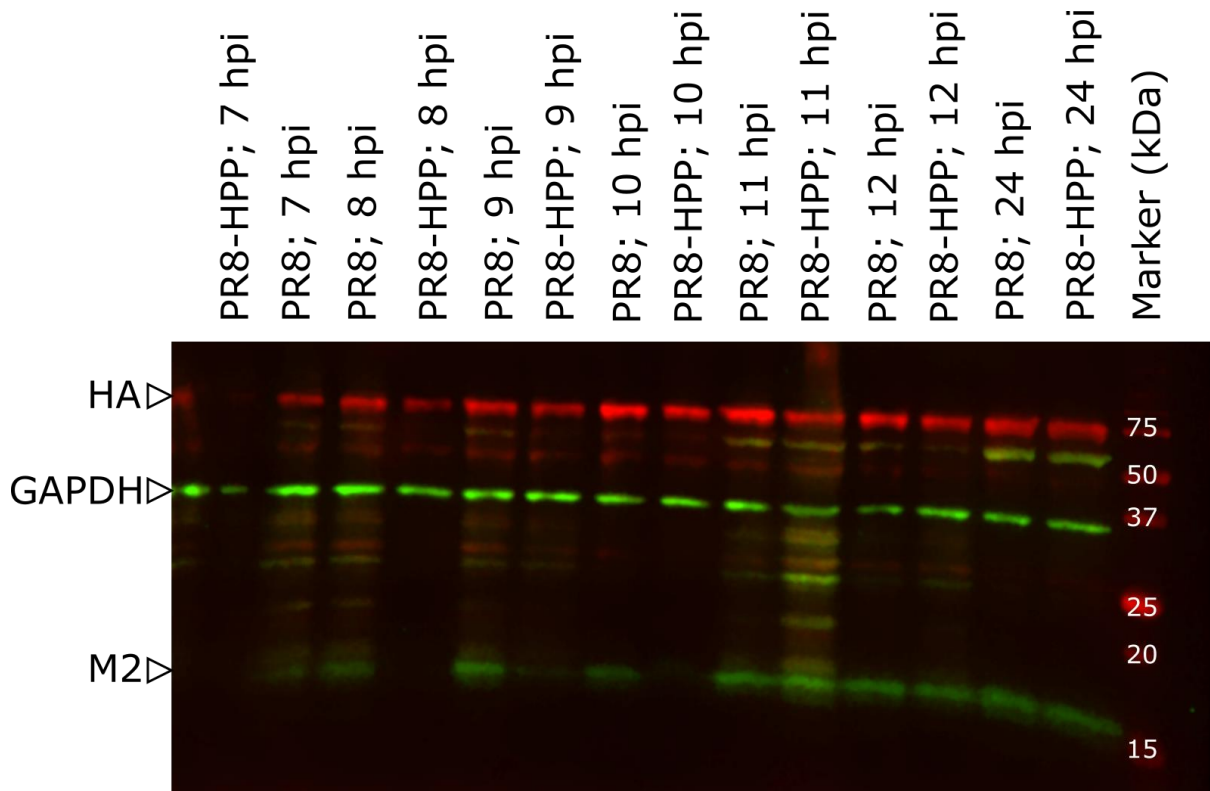

B

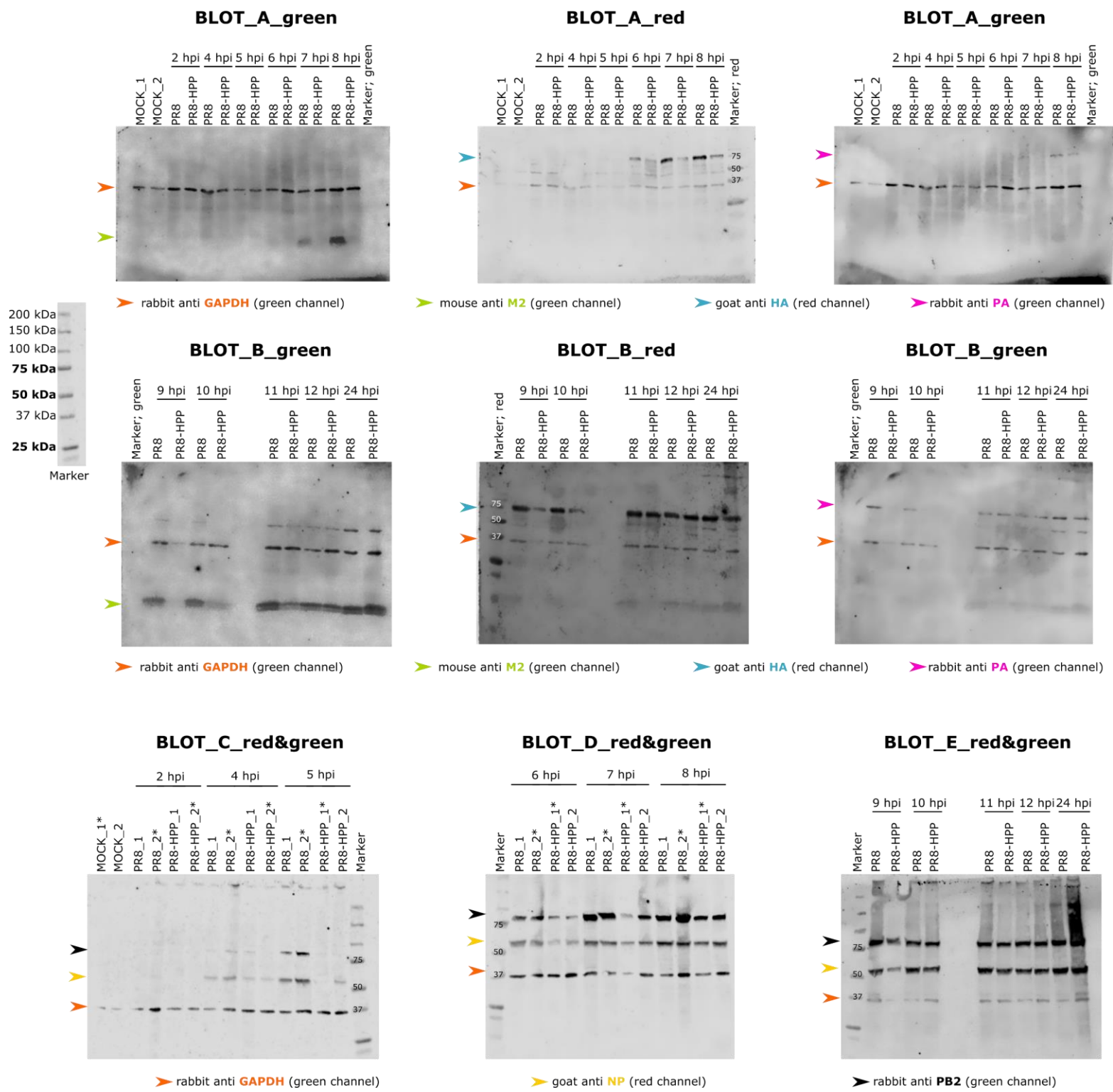

**C**

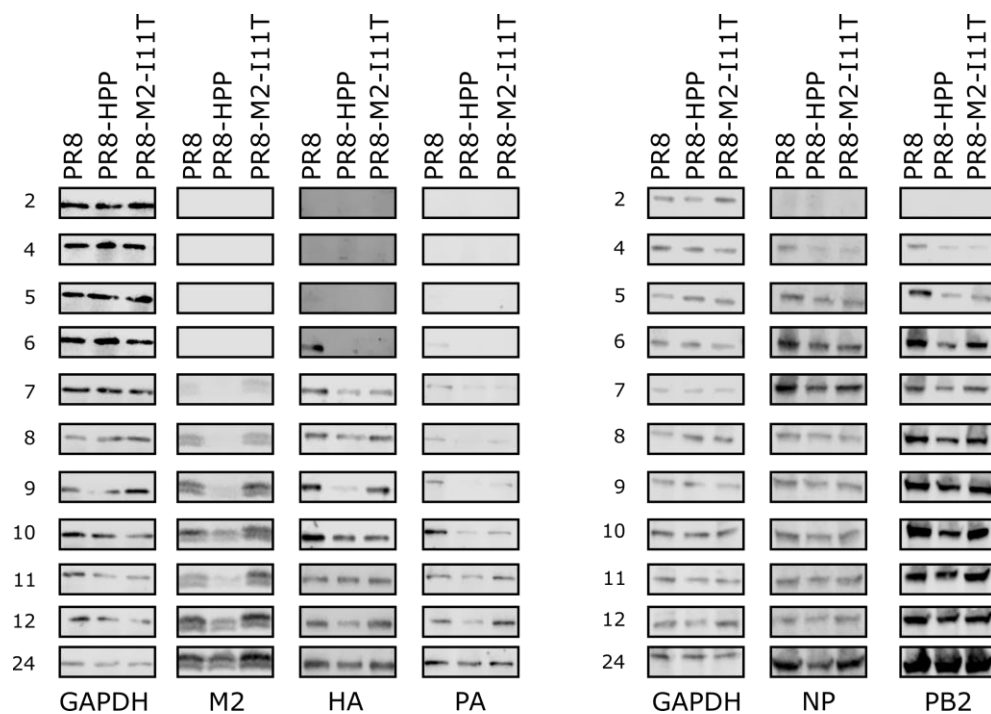

D

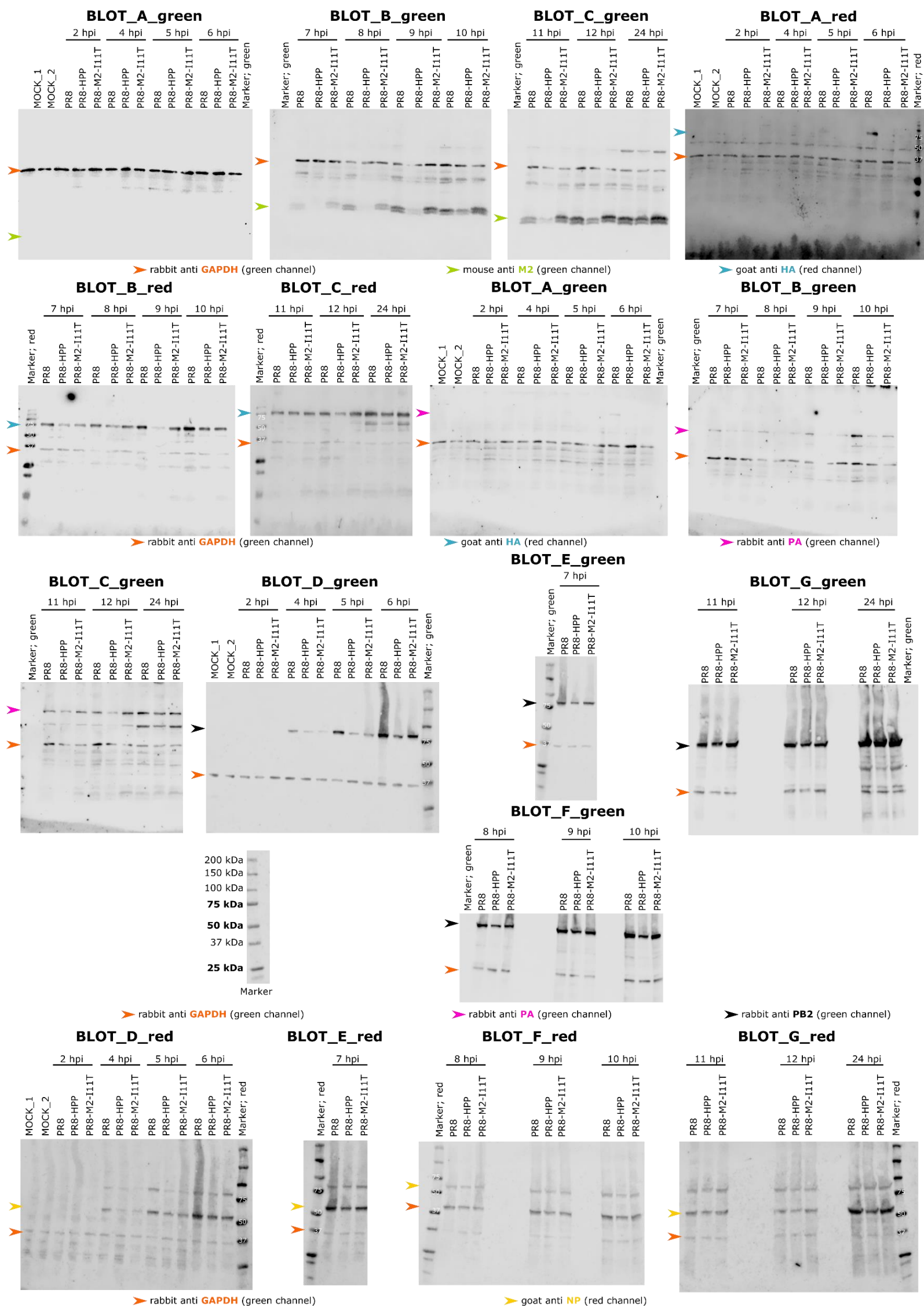

Supplement: FIG S3 [file mbio.00745-21-sf003.pdf]
