# Supplementary material for: In Vivo Therapy with M2e-Specific IgG Selects for an Influenza A Virus Mutant with Delayed Matrix Protein 2 Expression
Source: mBio. 2021 Jul 13;12(4):e00745-21. doi: 10.1128/mBio.00745-21 (PMC8406285; doi:10.1128/mBio.00745-21)
Supplement: TABLE S6 [file mbio.00745-21-st006.docx]

**Supplementary Table S6:** Variants detected above 10% in BAL fluid isolated from MAb 65-treated mice infected with 50 PFU of PR8, when mice lost 25% of their initial body weight.

| Experiment |  | Dpi | Segment | Position | Frequency | Amino acid change |
| --- | --- | --- | --- | --- | --- | --- |
| 2^nd^ | Mouse 1 | 37 | PB2 | 972 | 93.76 | PB2:p.Asp309Asn |
|  |  |  | PB2 | 1112 | 45.16 | PB2:p.Arg355Ser |
|  |  |  | PB1 | 157-158 | 11.89 | PB1:p.[Tyr38fs]; PB1-F2:p.[Thr7fs] |
|  |  |  | PA | 524 | 32.55 | Silent mutation |
|  |  |  | PA | 1165 | 95.69 | PA:p.[Met374Lys];  PA-N155:p.[Met220Lys];  PA-N182:p.[Met193Lys] |
|  |  |  | PA | 1712 | 95.55 | PA:p.[Gln556His];  PA-N155:p.[Gln402His];  PA-N182:p.[Gln375His] |
|  |  |  | HA | 497 | 22.57 | HA:p.Thr149Ala |
|  |  |  | HA | 501 | 10.28 | HA:p.Ala150Glu |
|  |  |  | HA | 765 | 91.15 | HA:p.Asp238Gly |
|  |  |  | HA | 771 | 31.66 | HA:p.Ala240Asp |
|  |  |  | HA | 834 | 31.44 | HA:p.Asn261Ser |
|  |  |  | HA | 1424 | 98.03 | HA:p.Val458Met |
|  |  |  | NP | 64 | 28.89 | Silent mutation |
|  |  |  | NA | 973 | 11.9 | Silent mutation |
|  |  |  | M | 762 | 99.96 | M2:p.[Pro10His] |
|  |  |  | M | 1014 | 11.22 | M2:p.[Ile94Thr] |
| 2^nd^ | Mouse 2 | 28 | PB2 | 1165 | 54.83 | PB2:p.Ile373Thr |
|  |  |  | PB2 | 1495 | 18.28 | PB2:p.Met483Thr |
|  |  |  | PB2 | 1527 | 53.78 | PB2:p.Val494Ile |
|  |  |  | HA | 823 | 96.38 | HA:p.Ile257Met |
|  |  |  | HA | 1424 | 98.85 | HA:p.Val458Met |
|  |  |  | NP | 1390 | 12.75 | NP:p.Thr442Ile |
|  |  |  | M | 762 | 90.99 | M2:p.[Pro10His] |
