# Supplementary material for: In Vivo Therapy with M2e-Specific IgG Selects for an Influenza A Virus Mutant with Delayed Matrix Protein 2 Expression
Source: mBio. 2021 Jul 13;12(4):e00745-21. doi: 10.1128/mBio.00745-21 (PMC8406285; doi:10.1128/mBio.00745-21)
Supplement: TABLE S7 [file mbio.00745-21-st007.docx]

**Supplementary Table S7:** Variants detected above 10% in BAL fluid isolated from MAb 65-treated mice infected with 100 PFU of PR8, when mice lost 25% of their initial body weight.

| Experiment |  | Dpi | Segment | Position | Frequency | Amino acid change |
| --- | --- | --- | --- | --- | --- | --- |
| 2^nd^ | Mouse 1 | 38 | PB2 | 1243 | 36.9 | PB2:p.Ile399Thr |
|  |  |  | PB2 | 1557 | 59.6 | PB2:p.Ile504Val |
|  |  |  | PB2 | 1570 | 37.57 | PB2:p.Arg508Gln |
|  |  |  | PB1 | 1025 | 15.36 | Silent mutation |
|  |  |  | PB1 | 1397 | 30.03 | Silent mutation |
|  |  |  | PB1 | 1583 | 15.01 | Silent mutation |
|  |  |  | PA | 1739 | 96.89 | Silent mutation |
|  |  |  | HA | 765 | 88.98 | HA:p.Asp238Gly |
|  |  |  | HA | 1217 | 85.99 | HA:p.Asn389Asp |
|  |  |  | HA | 1424 | 78.96 | HA:p.Val458Met |
|  |  |  | NP | 1037 | 29.96 | Silent mutation |
| 2^nd^ | Mouse 2 | 32 | PB2 | 972 | 13.75 | PB2:p.Asp309Asn |
|  |  |  | PB2 | 2265 | 89.13 | PB2:p.Asp740Asn |
|  |  |  | HA | 557 | 72.51 | HA:p.Glu169Lys |
|  |  |  | HA | 563 | 17.87 | HA:p.Glu171Lys |
|  |  |  | HA | 719 | 17.94 | HA:p.Asn223Asp |
|  |  |  | HA | 1003 | 21.76 | Silent mutation |
|  |  |  | HA | 1424 | 99.96 | HA:p.Val458Met |
|  |  |  | NP | 1195 | 67.07 | NP:p.Ser377Asn |
|  |  |  | M | 762 | 96.47 | M2:p.[Pro10His] |
|  |  |  | NS | 681 | 12.46 | NS1:p.[Pro212Leu]; NS2:p.[Leu55Phe] |
|  |  |  | NS | 703 | 26.74 | NS1:p.[Lys219Asn]; NS2:p.[Asn62Thr] |
